# Parkinson disease-associated mutations in LRRK2 cause centrosomal defects via Rab8a phosphorylation

**DOI:** 10.1186/s13024-018-0235-y

**Published:** 2018-01-23

**Authors:** Jesús Madero-Pérez, Elena Fdez, Belén Fernández, Antonio J. Lara Ordóñez, Marian Blanca Ramírez, Patricia Gómez-Suaga, Dieter Waschbüsch, Evy Lobbestael, Veerle Baekelandt, Angus C. Nairn, Javier Ruiz-Martínez, Ana Aiastui, Adolfo López de Munain, Pawel Lis, Thomas Comptdaer, Jean-Marc Taymans, Marie-Christine Chartier-Harlin, Alexandria Beilina, Adriano Gonnelli, Mark R. Cookson, Elisa Greggio, Sabine Hilfiker

**Affiliations:** 1 Institute of Parasitology and Biomedicine “López-Neyra”, Consejo Superior de Investigaciones Científicas (CSIC), Avda del Conocimiento s/n, 18016 Granada, Spain; 2 Department of Experimental Tumorbiology, Westfälische Wilhelms University Münster, Münster, Germany; 3 Laboratory for Neurobiology and Gene Therapy, KU Leuven, 3000 Leuven, Belgium; 4 Department of Psychiatry, Yale University School of Medicine, New Haven, USA; 5 Division of Neurosciences, Instituto Biodonostia-CIBERNED, San Sebastián, Spain; 6 Cell Culture Platform and Division of Neurosciences, Instituto Biodonostia, San Sebastián, Spain; 7 Division of Neurosciences, Instituto Biodonostia-CIBERNED, University of the Basque Country UPV-EHU, San Sebastián, Spain; 8 Medical Research Council Protein Phosphorylation and Ubiquitylation Unit, College of Life Sciences, University of Dundee, Dundee, Scotland DD1 5EH UK; 9 University of Lille, Inserm, CHU Lille, UMR-S 1172 - JPArc - Centre de Recherche Jean-Pierre AUBERT Neurosciences et Cancer, F-59000 Lille, France; 10 Cell Biology and Gene Expression Section, Laboratory of Neurogenetics, National Institute on Aging, National Institutes of Health, Bethesda, MD USA; 11 Department of Biology, University of Padova, 35131 Padova, Italy

**Keywords:** Centrosome, LRRK2, Parkinson’s disease, Phosphorylation, Rab8a

## Abstract

**Background:**

Mutations in LRRK2 are a common genetic cause of Parkinson’s disease (PD). LRRK2 interacts with and phosphorylates a subset of Rab proteins including Rab8a, a protein which has been implicated in various centrosome-related events. However, the cellular consequences of such phosphorylation remain elusive.

**Methods:**

Human neuroblastoma SH-SY5Y cells stably expressing wildtype or pathogenic LRRK2 were used to test for polarity defects in the context of centrosomal positioning. Centrosomal cohesion deficits were analyzed from transiently transfected HEK293T cells, as well as from two distinct peripheral cell types derived from LRRK2-PD patients. Kinase assays, coimmunoprecipitation and GTP binding/retention assays were used to address Rab8a phosphorylation by LRRK2 and its effects in vitro. Transient transfections and siRNA experiments were performed to probe for the implication of Rab8a and its phosphorylated form in the centrosomal deficits caused by pathogenic LRRK2.

**Results:**

Here, we show that pathogenic LRRK2 causes deficits in centrosomal positioning with effects on neurite outgrowth, cell polarization and directed migration. Pathogenic LRRK2 also causes deficits in centrosome cohesion which can be detected in peripheral cells derived from LRRK2-PD patients as compared to healthy controls, and which are reversed upon LRRK2 kinase inhibition. The centrosomal cohesion and polarity deficits can be mimicked when co-expressing wildtype LRRK2 with wildtype but not phospho-deficient Rab8a. The centrosomal defects induced by pathogenic LRRK2 are associated with a kinase activity-dependent increase in the centrosomal localization of phosphorylated Rab8a, and are prominently reduced upon RNAi of Rab8a.

**Conclusions:**

Our findings reveal a new function of LRRK2 mediated by Rab8a phosphorylation and related to various centrosomal defects.

**Electronic supplementary material:**

The online version of this article (10.1186/s13024-018-0235-y) contains supplementary material, which is available to authorized users.

## Background

Mutations in the *leucine-rich repeat kinase 2* (*LRRK2*) gene cause familial Parkinson’s disease (PD) and variations around the *LRRK2* locus increase risk for sporadic PD, indicating that abnormal LRRK2 function contributes to disease pathogenesis [1, 2]. Various pathogenic LRRK2 mutations have been described which all seem to converge on causing increased phosphorylation of select kinase substrates in intact cells [3], indicating that LRRK2 kinase activity may represent a therapeutic PD target. However, the downstream event(s) associated with abnormal LRRK2-mediated substrate phosphorylation remain unknown.

LRRK2 has been reported to be involved in a number of intracellular vesicular trafficking events [4–9] and also plays an important role in neurite outgrowth/cell polarity and cell migration [4, 10–14]. In dividing cells, pathogenic LRRK2 is known to impair neuronal precursor cell division in vitro and adult neurogenesis in vivo, deficits which may at least in part contribute to some of the age-dependent non-motor symptoms of PD patients [15–18]. LRRK2 is also highly expressed in various non-neuronal tissues, suggesting that it may play more general cellular role(s) shared amongst distinct cell types. Whilst displaying a broad subcellular distribution, LRRK2 can also partially localize to a centrosomal compartment [19]. Interestingly, a recent phosphoproteomics study has conclusively identified a subset of Rab proteins including Rab8a as LRRK2 kinase substrates [3]. Rab8a is a small GTPase localized to various intracellular compartments including Golgi, pericentrosomal recycling endosomes and centrosomes, and is known to regulate centrosome-related events [20–22]. However, the cellular consequences of LRRK2-mediated Rab8a phosphorylation are currently unknown.

Proper centrosome positioning is important for maintenance of cell polarity and directed migration [23–25]. The centrosome also plays an important role during the cell cycle, with centrosome duplication and separation allowing for the formation of a bipolar spindle required for appropriate chromosome segregation [26]. Finally, the centrosome plays a crucial role for membrane trafficking events to and from the pericentrosomal recycling endosome, and conversely, the pericentrosomal recycling endosome can modulate centrosomal maturation processes [20, 27], indicating that these two compartments cooperate to regulate various key cellular processes.

In the present study, we report that pathogenic LRRK2 causes alterations in centrosome positioning which are associated with deficits in neurite outgrowth and polarized cell migration. In dividing cells, pathogenic LRRK2 causes centrosomal cohesion deficits which are also observed in two distinct cell types derived from LRRK2-PD patients as compared to healthy controls, and are reverted by distinct LRRK2 kinase inhibitors. Furthermore, centrosomal cohesion and polarity deficits are observed when co-expressing wildtype LRRK2 along with wildtype but not phospho-deficient Rab8a mutant, and are associated with a kinase activity-dependent increase in the centrosomal accumulation of phosphorylated Rab8a. Finally, the centrosomal cohesion defects mediated by pathogenic LRRK2 are largely abolished upon RNAi of Rab8a. Altogether, these data indicate that pathogenic LRRK2 causes centrosomal alterations via Rab8a phosphorylation.

## Methods

### Cell culture and transfections

SH-SY5Y cells stably expressing GFP, flag-tagged wildtype LRRK2, or flag-tagged G2019S-mutant LRRK2 were cultured as described [28, 29]. Briefly, cells were grown in full medium (Dulbecco’s modified Eagle’s medium containing high glucose and 15% fetal bovine serum, non-essential amino acids, 50 μg/ml gentamycin (Life Technologies) and 200 μg/ml hygromycin B (Invivogen), and subcultured at a ratio of 1:6 twice a week. Transfection of cells was carried out at 80% confluence with 0.4 μg DNA and 1.5 μl Lipofectamine 2000 (Invitrogen) per well of a 24-well plate in 200 μl OptiMEM. Five h later, cells were changed into full medium, and passaged the following day at a ratio of 1:5 onto coverslips, followed by fixation and staining 72 h after transfection. For differentiation, 10,000 cells were plated onto coverslips in 24-well plates and grown in full medium for 24 h to allow for proper attachment. Cells were then changed to medium containing 3% fetal bovine serum and 10 μM retinoic acid (Sigma), and differentiated during 5 days, with medium changed every 48 h.

HEK293T cells were cultured as described [5, 8] and transfected at 80% confluence with 2 μg of LRRK2 constructs (and 200 ng of Rab constructs where indicated) and 6 μl of LipoD293 (SignaGen Laboratories) per well of a 6 well plate for 5 h in full medium. Cells were split to 20% confluence the following day, and processed for immunocytochemistry or Western blot analysis 24 h later.

HELA cells were cultured as previously described [8], Cells were plated in six-well plates at 40% confluence, and transfected the following day with 400 ng of GFP-tagged Rab8a constructs and 5 μl of Lipofectamine 2000 according to manufacturer’s instructions in full medium overnight. The following day, cells were split at a 1:4 ratio, plated onto coverslips, and processed 24 h later.

Primary human skin fibroblasts established from skin biopsies taken from five age- and sex-matched healthy control and five PD patients with the G2019S mutation [8], with informed consent and ethical approval, were grown in IMDM and 10% fetal bovine serum, with media changed every two days. Cells were subcultured at a ratio of 1:4, and seeded at equal densities on coverslips. All analyses were carried out on passages 3–8, and no passage-dependent differences were observed.

For lymphoblast generation, three healthy control and three PD patients due to the G2019S LRRK2 mutation were recruited at the Movement Disorders Unit of Lille University Medical Center (Lille, France, CPP Nord-Ouest 2008/09), with the two groups matched according to age and gender. Blood samples were collected in BD Vacutainer CPT Cell Preparation Tubes containing sodium heparin (Le Pont-de-Claix, France). The peripheral blood mononuclear cells (PBMCs) were collected and processed according to supplier’s recommendations. Lymphocytes were immortalized by infection with Epstein-Barr virus (EBV) as described [30]. Briefly, cell lines were established from freshly isolated lymphocytes or from cryopreserved lymphocytes using standard EBV transformation protocols that include cell separation by gradient centrifugation and lymphocyte growth enhancement by the mitogen phytohemagglutinin. Cell lines were maintained in RPMI 1640 medium supplemented with 20% fetal bovine serum, L-glutamine, 20 units/ml penicillin and 20 μg/ml streptomycin in T75 flasks in 5% CO_2_ at 37 °C. Cells were maintained at a density of 10^6^ cells/ml, with cell density monitored every other day using trypan blue staining.

Where indicated, cells were treated with nocodazole (200 nM, 2 h, SigmaAldrich), brefeldin A (5 μg/ml, 2 h, SigmaAldrich), or the indicated concentrations of MLi2 (MRC PPU, Dundee, UK), LRRK2-IN1 (obtained through the MJFF) or GSK2578215A (Tocris) before fixation.

### DNA constructs and site-directed mutagenesis

GFP-tagged human LRRK2 constructs were obtained from Addgene. Where required, mutations were introduced by site-directed mutagenesis (QuikChange, Stratagene). The GFP-G2019S-K1906 M kinase-dead LRRK2 construct was generated by site-directed mutagenesis introducing K1906 M on top of the G2019S construct, and the identity of constructs verified by sequencing of the entire coding region. DNA was prepared from bacterial cultures grown at 28 °C using a midiprep kit (Promega) according to manufacturer’s instructions. Human GFP-tagged Rab8a, Rab8a-Q67L and Rab8a-T22 N, as well as human GFP-tagged Rabin8 were obtained from Addgene. mRFP-tagged or triple-flag (3xFlag)-tagged Rab8a constructs were generated using Gibson Assembly Master Mix (New England Biolabs). Rab8a-T72A, Rab8a-T72D and Rab8a-T72E mutant constructs were generated by site-directed mutagenesis (QuikChange, Stratagene). The siRNA-resistant forms of mRFP-Rab8a and mRFP-Rab8a-T72A were generated by introducing 3 silent mutations into the target sequence of the seed region of the Rab8a siRNA (Silencer Select Rab8a (Ambion, ThermoFisher, ID s8679, nr 4,390,824). Specifically, the original sequence of the mRFP-Rab8a plasmids (5´-GCAAGAGAATTAAACTGCA-3′) was mutated to 5´-GCAAGAGAATTAAGTTACA-3′). Identity of all constructs was verified by sequencing of the entire coding region. HttQ74-GFP was a generous gift from D. Rubinsztein (Cambridge University, UK).

### Immunofluorescence and laser confocal imaging

HEK293T cells were fixed using 2% paraformaldehyde (PFA) in PBS for 10 min at room temperature, followed by permeabilization in MeOH for 2 min at − 20 °C. Cells were subsequently incubated in PBS containing 50 mM NH_4_Cl for 10 min [31], and then permeabilized with 0.2% Triton-X100/PBS for 20 min. For determination of the size of pericentrin-positive structures and for phospho-Rab8a staining, the MeOH and NH_4_Cl steps were omitted. HELA cells expressing GFP-tagged Rab8a constructs were fixed using 4% PFA in PBS for 15 min at room temperature, permeabilized with 0.5% Triton-X100/PBS for 5 min, rinsed in PBS, and mounted with mounting medium containing DAPI. SH-SY5Y cells were fixed using 2% paraformaldehyde (PFA) in PBS containing 4% sucrose for 20 min at room temperature, followed by permeabilization with 0.2% Triton-X100/PBS for 20 min. Primary fibroblasts were fixed with 2% paraformaldehyde (PFA) in PBS for 10 min at room temperature, followed by permeabilization with 0.2% Triton-X100/PBS for 20 min.

For lymphoblast immunocytochemistry, 13 mm diameter coverslips were placed into 24-well plates and coated with Cell-Tak Cell and Tissue Adhesive solution (Corning) following manufacturer’s protocols. After 20 min at room temperature, the solution was removed, coverslips were rinsed twice with distilled water and air-dried. Lymphoblast cells (200′000 per well) were added to dry coated coverslips, and cells attached by slight centrifugation at 690 g for 10 min at room temperature (without brake). Lymphoblast cells were fixed using 2% PFA in PBS for 20 min at room temperature, followed by permeabilization with 0.2% Triton-X100/PBS for 20 min at room temperature.

After fixation and permeabilization, coverslips were blocked for 1 h with 0.5% (*w*/*v*) BSA in 0.2% Triton-X100/PBS (blocking solution), followed by incubation with primary antibodies in blocking solution overnight at 4°C. Primary antibodies included rabbit polyclonal anti-pericentrin (Abcam, ab4448, 1:1000), mouse monoclonal anti-pericentrin (Abcam, ab28144, 1:1000), mouse monoclonal anti-γ-tubulin (Abcam, ab11316, 1:1000), mouse monoclonal anti-c-Myc (Sigma, clone 9E10, M4439 1:500), mouse monoclonal anti-flag (Sigma, clone M2, 1:500), rabbit polyclonal anti-Rab8a (Millipore, ABC423, 1:1000), mouse anti-Golgin97 (Molecular Probes, A-21270, 1:100), rabbit polyclonal anti-β-COP (Invitrogen, PA1–061, 1:750), and mouse monoclonal p230/Golgin-245 (Becton Dickinson, 611,280, 1:400). The sheep anti-Rab8a and anti-T72-phospho-Rab8a antibodies have been previously described (MRC PPU, S969D and S874D, respectively) [3]. For the sheep anti-Rab8a antibody, the NH_4_Cl step was omitted, and for the sheep anti-T72-phospho-Rab8a antibody, both the MeOH and NH_4_Cl steps were omitted. Sheep antibodies were used at a 1:50 dilution, and the anti-T72-phospho-Rab8a antibody was used in the presence of a 10-fold molar excess of dephospho-peptide, or of phospho-peptide where indicated. Importantly, all double- and triple-immunocytochemistry involving sheep antibodies were performed sequentially, with the sheep antibodies employed first.

Secondary antibodies included Alexa 405-conjugated goat anti-mouse or goat anti-rabbit, Alexa 488-conjugated goat anti-mouse or goat anti-rabbit, Alexa 594-conjugated goat anti-mouse or goat anti-rabbit, Alexa 633-conjugated goat anti-mouse or goat anti-rabbit (Invitrogen, 1:1000), Alexa 488-conjugated donkey anti-sheep (Invitrogen, 1:1000) or Alexa 594-conjugated donkey anti-sheep (Abcam, 1:1000). As indicated, cells were either mounted using mounting medium containing DAPI (Vector Laboratories), or incubated with TO-PRO-3 Iodide (642/661) (Invitrogen, 1:1000) for 3 min, followed by washes in PBS before mounting in ProLong Gold Antifade mounting medium (Invitrogen).

Images were acquired on a Leica TCS-SP5 confocal microscope using a 63X 1.4 NA oil UV objective (HCX PLAPO CS). Images were collected using single excitation for each wavelength separately and dependent on secondary antibodies (405 nm UV diode and a 415–455 nm emission band pass; 488 nm Argon Laser line and a 510–540 nm emission band pass; 543 HeNe Laser line and a 600–630 nm emission band pass; 633 HeNe Laser line and a 640–670 nm emission band pass). GFP-tagged proteins were excited with 488 nm Argon Laser line and a 500–530 nm emission band pass, and RFP-tagged proteins with 543 nm HeNe Laser line and a 560–590 nm emission band pass, respectively. DAPI was excited with the 405 nm UV diode and a 430–480 nm emission band pass, and TO-PRO with 633 nm HeNe Laser line and a 650–675 nm emission band pass, respectively.

10–15 image sections of selected areas were acquired with a step size of 0.5 μm, and z-stack images analyzed and processed using Leica Applied Systems (LAS AF6000) image acquisition software. The same laser intensity settings and exposure times were used for image acquisition of individual experiments to be quantified. For quantification of centrosome size, a circle was drawn around individual centrosomes and area quantified using image acquisition software as described above, where mature centrosomes were around 1 μm^2^, and immature centrosomes around 0.5 μm^2^. Centrosomes were scored as being separated when the distance between their centers was > 1.5 μm (for HEK293T and SH-SY5Y cells) as analyzed by ImageJ software. For fibroblasts, the mean distance between separated centrosomes in control cells was 2.25 ± 0.2 μm (mean ± s.e.m., *n* = 10 cells), and centrosomes scored as being separated when the distance was > 2.5 μm. Using equivalent criteria for lymphoblasts, centrosomes were scored as being separated when the distance was > 1.3 μm. In all cases, mitotic cells were excluded from this analysis.

Quantification of the phospho-Rab8a signal in SH-SY5Y cells was done over non-processed and non-saturated images acquired during the same day with the same laser intensities. Quantification was performed with Leica Applied Systems (LAS AF6000) image acquisition software. Circular ROIs of 2.2 μm width and 2.2 μm height were set on top of the centrosomal signal, and mean intensity from the phospho-Rab8a signal obtained from at least 50 cells per condition and experiment. Background signal was subtracted in all cases by placing the ROI in a different aleatory place within the same cell. Similarly, to quantify phospho-Rab8a signals in lymphoblasts, a circle of 3 μm diameter was drawn around individual centrosomes as assessed by pericentrin staining, and the phospho-Rab8a fluorescence intensity from around 30–50 individual cells quantified from maximal intensity projections using Leica Applied Systems (LAS AF6000) image acquisition software.

Most experiments were quantified by two independent observers, and some experiments were quantified by a third observer blind to condition, with similar results obtained in all cases.

### Knockdown of Rab8a by RNA interference

HEK293T cells were seeded in 6-well plates at 30–40% confluence one day prior to transfection such that they were at a confluence of 70–80% the following day. They were transfected with 2 μg of GFP-LRRK2 DNA and 25 nM siRNA using 4 μl of jetPRIME Transfection Reagent (Polyplus-Transfection SA, no 114–15) in 200 μl jetPRIME buffer. The mix was incubated for 15 min at room temperature and added to 2 ml of full medium per well of a 6-well plate overnight. For knockdown experiments in the presence of both GFP-LRRK2 and mRFP-Rab8a or mRFP-Rab8a-T72A (sensitive or resistant to siRNA, respectively), cells were transfected with 50 nM of the indicated siRNA using 4 μl of jetPRIME Transfection Reagent as described above. Four hours later, media was replaced and cells transfected with 2 μg of the indicated LRRK2 constructs and 200 ng of the indicated Rab8a constructs and 6 μl of LipoD293 (SignaGen Laboratories) per well of a 6-well plate overnight in full medium. In all cases, cells were passaged 24 h later and processed for Western blot analyis or immunocytochemistry 48 h after transfection. RNAi reagents included Silencer Select Negative Control no. 1 siRNA (Ambion, ThermoFisher, cat. nr 4,390,843) and Silencer Select Rab8a (Ambion, ThermoFisher, ID s8679, cat. nr 4,390,824).

For determination of Rab8a levels, cells were washed in PBS and resuspended in cell lysis buffer (1% SDS in PBS containing 1 mM PMSF, 1 mM Na_3_VO_4_, 5 mM NaF). Extracts were sonicated, boiled and centrifuged at 13,500 rpm for 10 min at 4 °C. Protein concentration of supernatants was estimated using the BCA assay (Pierce), and 30 μg of extracts resolved by SDS-PAGE and analyzed by Western blotting.

### Wound healing and cell migration assays

For wound-healing assays, 40,000 SH-SY5Y cells were seeded on each side of 35 mm insert-containing dishes (IBIDI) and grown to confluence in full medium. The wound (500 μm diameter) was generated by removing the insert, cells were gently washed three times in full medium, and phase contrast images acquired every 10 min for 15 h on a Leica TCS-SP5 confocal microscope using a 10X objective (C-PLAN 10.0 × 0.22 POL) and 1.3X zoom. Overall wound healing migration speed was calculated as average speed of the cell front using ImageJ software analysis. For single cell tracking, individual cells in the first row facing the wound were tracked (ImageJ manual tracking plugin) until reaching the middle of the wound [32], and individual cell migration speed, directionality (D) and forward migration index in Y (FMI Y) calculated from at least 30 cells per condition and experiment using the Chemotaxis and Migration Tool (IBIDI).

For determination of cell polarity, cells were processed for immunocytochemistry 4 h after generating the wound using anti-pericentrin and anti-golgin97 antibodies as described above. The first row of cells facing the wound was analyzed, and cells were scored as polarized when located in a 90° sector emerging from the center of the nucleus and facing the wound edge [33]. Basal levels of expected random orientation of 25% were confirmed by analyzing centrosome orientation immediately after generating the wound.

### Cell extracts and western blotting

HEK293T cells were collected 48 h after transfection, washed in PBS and resuspended in cell lysis buffer (1% SDS in PBS containing 1 mM PMSF, 1 mM Na_3_VO_4_, 5 mM NaF). Extracts were sonicated, boiled and centrifuged at 13,500 rpm for 10 min at 4 °C. Protein concentration of supernatants was estimated using the BCA assay (Pierce), and 40 μg of extracts resolved by SDS-PAGE and analyzed by Western blot, using a rabbit polyclonal anti-GFP antibody (ab6556, 1:3000, Abcam), phospho-S935-LRRK2 antibody (ab133450, Abcam, 1:500), a mouse monoclonal anti-myc antibody (clone 9E10, 1:1000, Sigma), a sheep polyclonal anti-Rab8a (MRC PPU, S969D, 1:200), a sheep polyclonal anti-phospho-Rab8a (MRC PPU, S874D, 1:200), a mouse monoclonal anti-flag antibody (clone M2, 1:2000, Sigma) and a mouse monoclonal anti-tubulin antibody (clone DM1A, 1:10,000, Sigma) as loading control.

For lymphoblast cell extracts, 10^6^ cells were centrifuged at 1030 g for 10 min at 4 °C. The pellet was resuspended in 100 μl of lysis buffer (20 mM Tris-HCl pH 7.4, 150 mM NaCl, 1 mM EDTA, 10% glycerol, 1% Triton-X100) containing protease and phosphatase inhibitor cocktails (SigmaAldrich), and incubated for 30 min on a rotary wheel at 4 °C. Cell extracts were centrifuged at 13,500 rpm for 5 min at 4 °C, and supernatants quick-frozen in liquid N_2_ and stored at − 80 °C. Protein concentration of supernatants was estimated using the BCA assay (Pierce), and 20 μg of extracts resolved by SDS-PAGE and analyzed by Western blot, using a rabbit polyclonal phospho-S935-LRRK2 antibody (ab133450, Abcam, 1:500), a mouse monoclonal anti-LRRK2 antibody (UC Davies/NIH NeuroMab clone 75–253, 1:1000), a sheep polyclonal anti-Rab8a (MRC PPU, S969D, 1:200) and a mouse monoclonal anti-tubulin antibody (clone DM1A, 1:10,000, Sigma) as loading control. Some Westerns were developed with ECL reagents (Roche), and a series of timed exposures to ensure that densitometric analyses were performed at exposures within the linear range. Quantification was performed using QuantityOne software (BioRad). Western blotting of phospho-S935 and total LRRK2 in extracts from lymphoblasts was performed with ECL Prime Western Blotting Detection Reagent (GEHealthcare), and analysis in Amersham Imager 600 (GEHealthcare). The majority of Western blotting was performed according to the protocol described by LI-COR for Near-Infrared Western Blot Detection. In all cases, incubation with primary antibodies was performed overnight at 4 °C, and secondary antibodies were incubated for 1 h at RT. For analysis of Rab8a or phospho-Rab8a levels using this technology, a rabbit monoclonal anti-Rab8a antibody (ab188574, Abcam, 1:1000), and a rabbit polyclonal phospho-Rab8a antibody were employed. Blots were imaged using an Odyssey CLx system, and quantification was done using the instrument’s Image Studio software.

### Immunoprecipitation of GFP-Rabin8

HEK293T cells were cultured as described and co-transfected at 80% confluence with 600 ng of GFP-Rabin8 and 200 ng of flag-tagged Rab8a constructs using 6 μl of LipoD293 (SignaGen Laboratories) per well of a 6 well plate overnight in full medium. The following day, cells were split into 100 mm tissue culture plates, and were collected 48 h after transfection. Cells were washed in PBS, followed by resuspension in 1 ml of lysis buffer (50 mM Tris-HCl, pH 7.6, 150 mM NaCl, 2 mM MgCl_2_, 1% Triton-X100, 1 mM DTT, protease inhibitor cocktail (Roche) and phosphatase inhibitor cocktail 3 (Sigma)), and incubated on a rotary wheel for 1 h at 4 °C. Lysates were subsequently spun at 13′000 rpm for 10 min at 4 °C, and protein concentration of supernatants estimated by BCA assay (Sigma), with 1 mg of total protein subjected to immunoprecipitation with a rabbit polyclonal anti-GFP antibody (Abcam, Ab 6556, 1 μg per sample). Lysates were incubated with antibody for 2 h at 4 °C, followed by addition of protein G Sepharose Fast Flow (Amersham) and incubation overnight at 4 °C. The next day, beads were washed three times with lysis buffer, and bound proteins eluted with Laemmli sample buffer and heating at 95 °C for 4 min prior to separation by SDS-PAGE and Western blotting as indicated above, using a mouse monoclonal anti-GFP antibody (Roche, 1:1000) or a mouse monoclonal anti-flag antibody (clone M2, 1:1000, Sigma), respectively.

### Cell transfection, protein purification and in vitro kinase assays

HEK293T cells were cultured in Dulbecco’s modified Eagle’s medium (DMEM) supplemented with 10% (*v*/v) fetal bovine serum, penicillin (100 units/ml) and streptomycin (100 μg/ml) (Life Technologies) at 37 °C and 5% CO_2_. 3xFlag-LRRK2 (wildtype and G2019S mutant) and 3xFlag-Rab8a (wildtype and T72A mutant) were transiently transfected using linear polyethylenimine (PEI, Polyscience) with a 1:2 DNA:PEI ratio. The transfection mixes were prepared by dissolving 40 μg of DNA in 1 ml of OPTI-MEM with 80 μl of PEI (40 μM final). Mixes were incubated at room temperature for 20 min and subsequently added to HEK293T cells previously plated on a 15 cm^2^ Petri dish.

Seventy-two hours after transfection, cells were resuspended in 1 ml of lysis buffer (10 mM Tris-HCl pH 7.5, 150 mM NaCl, 5 mM EDTA, 2.5 mM Na_4_P_2_O_7_, 1 mM beta-glycerophosphate, 1 mM Na_3_VO_4_, supplemented with protease inhibitor mixture (Sigma-Aldrich) and 1% (*v*/v) Tween-20). Samples were left on ice for 30 min, and cell lysates collected after centrifugation at 18000×g for 35 min at 4 °C. Supernatants were collected and incubated overnight with 40 μl of Anti-Flag M2 Affinity Gel beads (Sigma-Aldrich) at 4 °C with mild agitation. Beads were pelleted and washed with 1 ml of five different wash buffers (WB): WB1 (20 mM Tris-HCl pH 7.5, 500 mM NaCl, 1% (*v*/v) Tween-20) twice, WB2 (20 mM Tris-HCl pH 7.5, 350 mM NaCl, 1% (v/v) Tween-20) twice, WB3 (20 mM Tris-HCl pH 7.5, 150 mM NaCl, 1% (v/v) Tween-20) twice, WB4 (20 mM Tris-HCl pH 7.5, 150 mM NaCl, 0.1% (v/v) Tween-20) twice and WB5 (20 mM Tris-HCl pH 7.5, 150 mM NaCl, 0.02% (*v*/v) Tween-20) once. All 3xFlag-tagged proteins were eluted in elution buffer (25 mM Tris-HCl pH 7.5, 5 mM beta-glycerophosphate, 2 mM DTT, 0.1 mM Na_3_VO_4_, 10 mM MgCl_2_ supplemented with 0.007% Tween-20, 150 ng/μl 3xFlag peptide (Sigma-Aldrich) for subsequent in vitro kinase assays.

For experiments in which Rab8a was loaded with specific guanine nucleotides, affinity resin bound-protein was washed as above, rinsed in loading buffer (20 mM Tris pH 7.5, 150 mM NaCl, 5 mM EDTA, 0.02% (*v*/v) Tween-20) and incubated with an excess (200 μM) GDP or GTPγS for 30 min at 30 °C with slight agitation. Nucleotide exchange was stopped and excess nucleotides removed by rinsing beads three times in kinase buffer (25 mM Tris-HCl pH 7.5, 10 mM MgCl_2_, 2 mM dithiothreitol (DTT), 0.02% (v/v) Tween-20, 5 mM beta-glycerophosphate, 0.1 mM Na_3_VO_4_) [34] followed by elution with kinase buffer supplemented with 150 ng/μl 3xFlag peptide.

For in vitro kinase assays, purified wildtype or T72A-mutant 3xFlag-Rab8a proteins eluted in kinase buffer, with and without nucleotide loading, were incubated with wildtype or G2019S mutant 3xFlag-LRRK2 at a ratio of 50:1 (Rab8a:LRRK2). Reactions were kept at 30 °C for 1 h in the presence of ATP-γ^33^P (1 μCi/reaction) and 2.5 μM cold ATP and then stopped by adding Laemmli buffer (100 mM Tris-HCl pH 6.8, 4% (*w*/*v*) SDS, 200 mM DTT, 20% (*v*/v) glycerol and Bromophenol Blue). Incorporated ^33^P was detected by autoradiography with a Phospho-Imager system (Cyclone, Perkin-Elmer). The same membranes were stained with Coomassie Blue or probed with an anti-Flag antibody for total protein loading, and quantification performed using ImageJ software.

### Rab8a GTP binding and GTP retention assays

HEK293T cells were transfected with 3xFlag-Rab8a wildtype or mutant plasmids as indicated and 24 h later cells were lysed in buffer containing 20 mM Tris-HCl pH 7.5, 150 mM NaCl, 1 mM EDTA, 1% Triton X-100, 10% glycerol and protease inhibitor cocktail (Roche) for 30 min on ice. Lysates were centrifuged (10 min, 20,000×*g*), supernatants were precleared with Easy view Protein G agarose (Sigma-Aldrich) for 30 min at 4 °C, followed by incubation with anti-Flag M2 agarose beads (Sigma-Aldrich) for 1 h at 4 °C with mild agitation. Proteins bound to beads were washed 4 times with 25 mM Tris-HCl pH 7.5, 400 mM NaCl, and 1% Triton X-100.

For GTP or GDP binding assays, equal amounts of wildtype or mutant 3xFlag-Rab8a fusion proteins bound to anti-Flag M2 agarose beads (Sigma-Aldrich) were washed twice with Buffer A (20 mM Tris–HCl pH 7.5, 100 mM NaCl, 5 mM MgCl_2_, 1 mM NaH_2_PO_4_, 2 mM DTT) and incubated overnight on ice in Buffer A containing 0.1 μM ^3^H–GTP or ^3^H–GDP. Beads were then washed twice in Buffer A to remove unbound nucleotides, added to Bio-safe II (RPI) scintillation cocktail, and binding quantified using scintillation counting for H^3^ (Tri-Carb 2810TR scintillation counter, Perkin Elmer).

To assay GTP or GDP retention, agarose-bound Rab8a proteins were incubated in Buffer A containing 0.1 μM ^3^H–GTP or ^3^H–GDP overnight on ice and washed twice with Buffer A to remove unbound nucleotide. Subsequently, proteins were incubated in Buffer A containing a 100-fold excess of unlabeled GTP or GDP for 0, 15, 30 or 60 min, and shaking at 37 °C. After each time point, Rab8a proteins were washed twice with Buffer A, and retained ^3^H–GTP or ^3^H–GDP bound to proteins was quantified using scintillation counting. The amount of ^3^H–GTP or ^3^H–GDP bound at 15, 30 and 60 min for each sample was calculated as a fraction of initial binding.

### Co-immunoprecipitation

HEK293FT cells transfected with 3xflag-Rab8a plasmids were lysed in IP buffer (20 mM Tris-HCl pH 7.5, 150 mM NaCl, 1 mM EDTA, 0.3% Triton X-100, 10% Glycerol, and protease inhibitor cocktail (Roche)) for 30 min on ice. Lysates were centrifuged at 4 °C for 10 min at 20,000 g and supernatants were further cleared by incubation with Easy view Protein G agarose beads (Sigma) for 30 min at 4 °C. After agarose bead removal by centrifugation, lysates were incubated with anti-flag M2 agarose beads (Sigma) for 1 h at 4 °C with mild agitation. Beads were washed three times with IP wash buffer (20 mM Tris-HCl pH 7.5, 150 mM NaCl, 1 mM EDTA, 0.1% Triton X-100, 10% Glycerol) and eluted in buffer containing 20 mM Tris-HCl, 100 mM NaCl, 5 mM MgCl_2_ and 150 ng/μl of 3xflag peptide by shaking for 15 min at 4 °C. Each co-immunoprecipitation was repeated in 3 independent experiments, and samples analyzed by Western blotting using an anti-GDI1/2 antibody (1:2000, Life Technologies, 710,300) or an anti-flag antibody (1:500, Sigma, F3165).

### Mass spectrometry

Purified wildtype and mutant Rab8a proteins were separated on 4–20% SDS-PAGE, stained with Coomassie brilliant blue staining (Thermo Scientific) and a band corresponding to a ~ 50 kDa protein was excised. Protein identification was performed using MASCOT.

### Statistical analysis

All data are expressed as means ± s.e.m. Unless otherwise noted, data were analyzed by one-way ANOVA with Tukey’s *post-hoc* test, and *p* < 0.05 was considered significant. Statistical details to all experiments can be found in the figure legends. ***p < 0.005; **p < 0.01; *p < 0.05.

## Results

### Pathogenic LRRK2 causes deficits in centrosomal positioning critical for cell polarization and directed migration

**Fig. 1:**
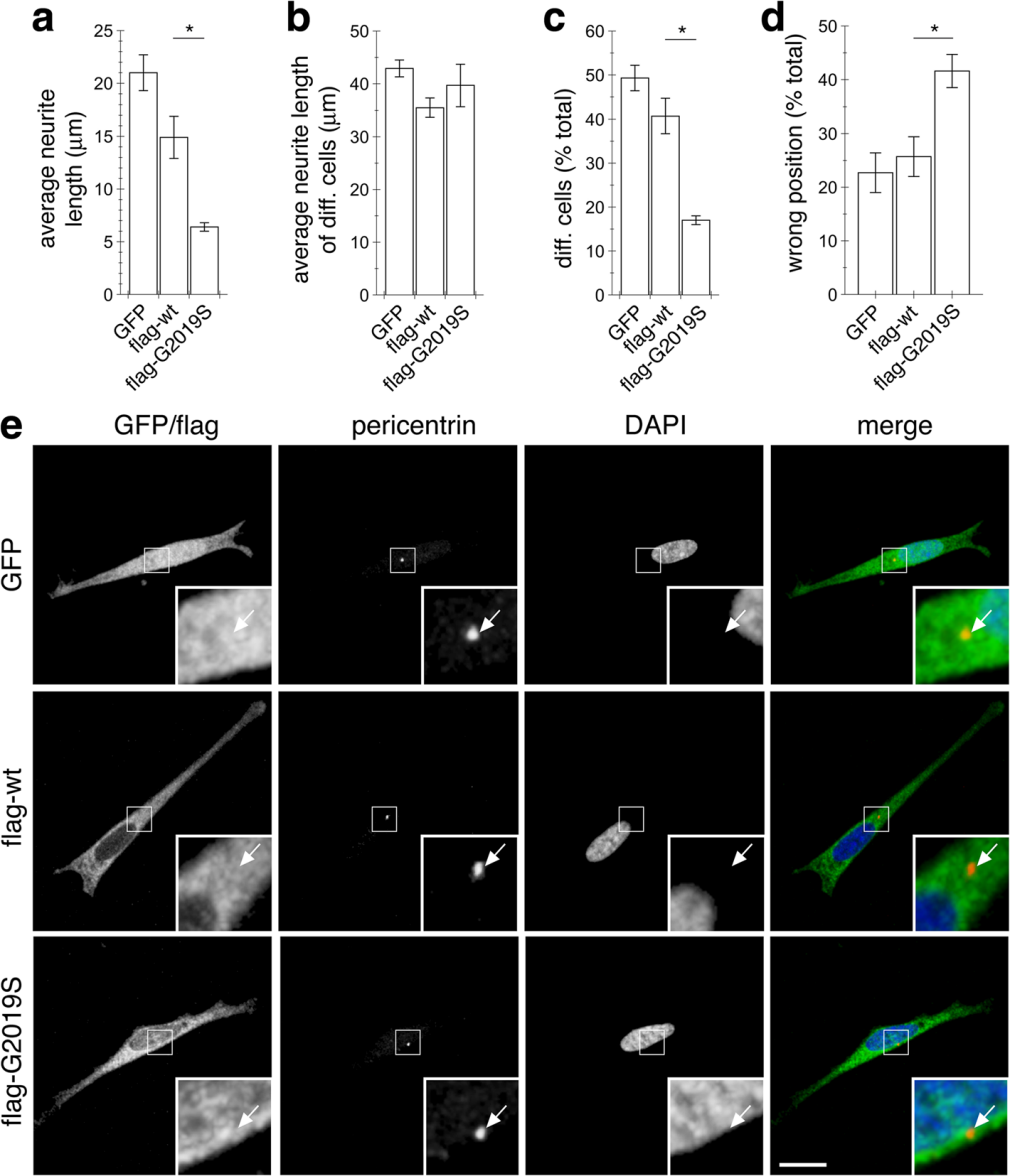
Pathogenic LRRK2 causes deficits in differentiation and altered centrosome positioning in differentiated SH-SY5Y cells. **a** Quantification of neurite length in SH-SY5Y cells differentiated with retinoic acid for 5 days. Around 150 cells were analyzed per condition and experiment. Bars represent mean ± s.e.m., (*n* = 3 independent experiments); *, *p* < 0.05. **b** Quantification of neurite length only including differentiated cells. Around 150 differentiated cells were analyzed per condition and experiment. Bars represent mean ± s.e.m., (*n* = 3 independent experiments). **c** Quantification of percentage of differentiated cells. Bars represent mean ± s.e.m., (*n* = 3 independent experiments); *, *p* < 0.05. **d** Quantification of differentiated cells where centrosome is positioned on the side or opposite the longest neurite in the distinct cells as indicated. Around 30 non-contiguous differentiated cells were analyzed per condition and experiment. Bars represent mean ± s.e.m., (*n* = 3 independent experiments); *, *p* < 0.05. **e** Example of non-contiguous SH-SY5Y cells stably expressing GFP, or flag-tagged wildtype or G2019S-mutant LRRK2 as indicated, and stained for pericentrin and DAPI. Scale bar, 10 μm

To evaluate effects of pathogenic LRRK2 on cell polarity, we employed human neuroblastoma SH-SY5Y cells stably expressing flag-tagged wildtype or G2019S mutant LRRK2 [28, 29]. Cells were differentiated with retinoic acid, and average neurite length quantified. Pathogenic G2019S LRRK2-expressing cells showed a significant decrease in the percentage of differentiated cells as compared to control or wildtype LRRK2-expressing cells (Fig. 1a-c). Since proper positioning of the centrosome is important for cell polarization and directed migration [24, 25], we analyzed centrosome positioning from non-contiguous differentiated cells by staining for pericentrin, a component of the pericentriolar matrix [35]. The centrosome was facing the longest neurite in the majority of control and wildtype LRRK2-expressing cells, whilst a significant amount of G2019S-expressing cells had their centrosome positioned on the side, or opposite the longest neurite (Fig. 1d and e).

**Fig. 2:**
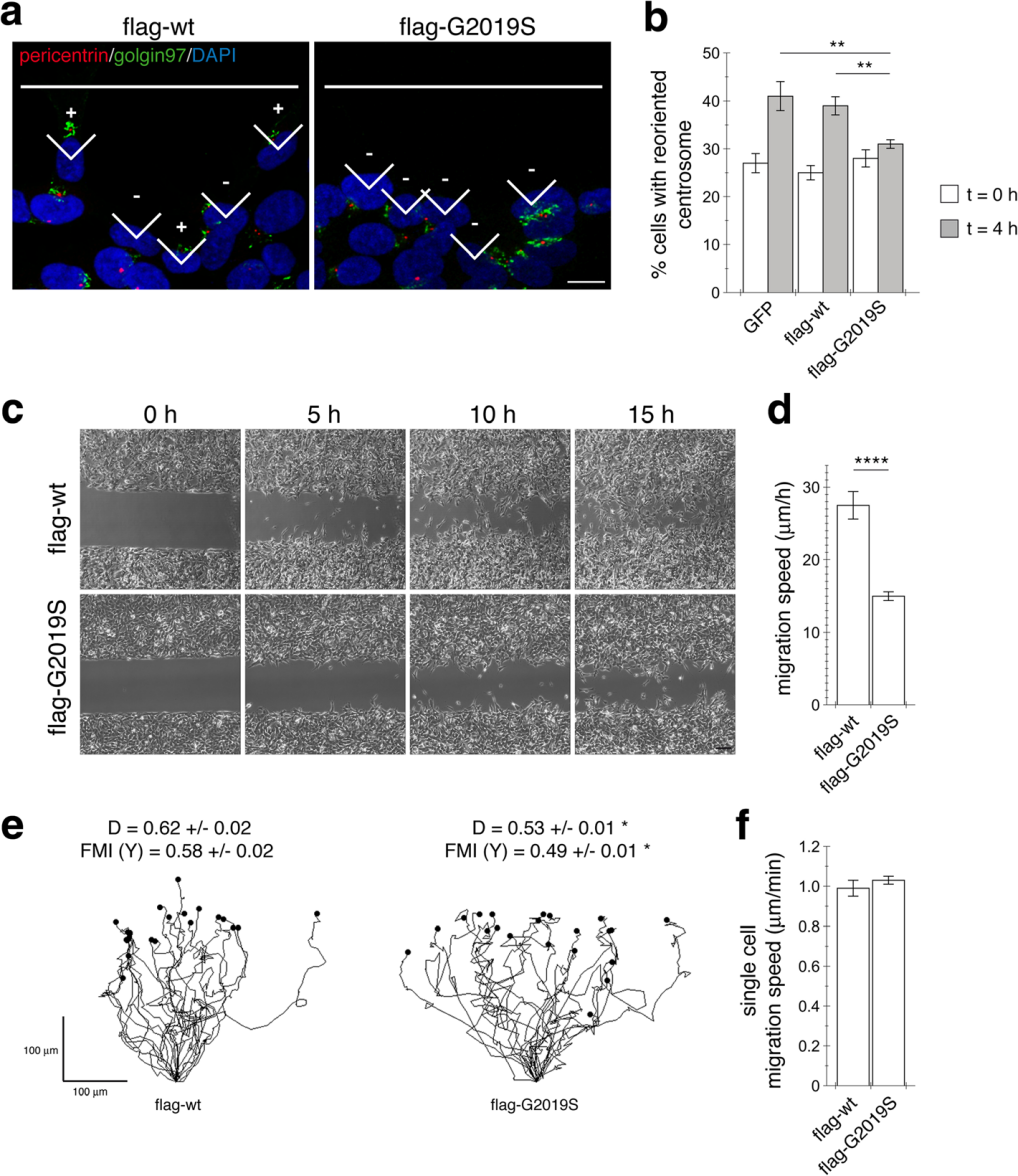
Pathogenic LRRK2 causes deficits in centrosomal positioning critical for cell polarization and directed migration. **a** Example of reorientation of the centrosome 4 h after wounding in SH-SY5Y cells stably expressing flag-tagged wildtype or G2019S mutant LRRK2. The white lines indicate scratch orientation, and cells were stained with anti-pericentrin, anti-Golgin97, and DAPI. Angles are labeled as having oriented (+) or not oriented (−) centrosomes for the first row of cells facing the scratch wound. Scale bar, 10 μm. **b** Quantification of centrosome reorientation in cells stably expressing GFP, flag-tagged wildtype or G2019S-mutant LRRK2 immediately after (*t* = 0 h), or 4 h after generating the wound (*t* = 4 h). Random orientation is expected to be 25%. *N* > 100 cells were quantified for each condition in each experiment. Bars represent mean ± s.e.m. (*n* = 3 independent experiments); **, *p* < 0.01. **c** Wound-healing assays in either flag-tagged wildtype or G2019S-mutant LRRK2-expressing SH-SY5Y cells. Phase-contrast images at the indicated times are shown. Scale bar, 150 μm. **d** Quantitative analysis of wound-healing assays as described in Methods. Bars represent mean ± s.e.m. (*n* = 6 independent experiments); ****, *p* < 0.001. **e** Example of tracking of individual cells expressing flag-tagged wildtype or G2019S mutant LRRK2. Individual cells in the first row facing the wound were tracked until reaching the middle of the wound. Directionality index (D) and forward migration index in Y axis (FMI Y) were calculated for at least 30 cells per condition in three independent experiments, and are expressed as mean ± s.e.m. on top of each graph. *, *p* < 0.05. **f** Single cell wound healing migration speed was calculated for at least 30 cells per condition in 3 independent experiments

As cell polarization is a prerequisite for cell migration [25], we wondered whether pathogenic LRRK2-expressing cells display deficits in cell migration associated with altered centrosome positioning. Scratch wounding was performed on control GFP, wildtype or G2019S mutant LRRK2-expressing SH-SY5Y cells, and cells counted as oriented when the centrosome was located within a 90° angle facing the wound [32, 33, 36] (Fig. 2a). After 4 h of wounding, around 40% of control GFP and wildtype LRRK2-expressing cells had already reoriented the centrosome, whilst the percentage of reoriented centrosomes was significantly less in G2019S LRRK2-expressing cells (Fig. 2b). To test for potential migration defects, cells were recorded by live cell microscopy after performing a scratch wound (Fig. 2c and d). G2019S-LRRK2-expressing cells displayed a decrease in cell migration in the wound healing assay as compared to wildtype LRRK2-expressing cells. Whereas wildtype LRRK2-expressing cells exhibited persistent directional migration until the closure of the gap, G2019S-LRRK2-expressing cells displayed less directional migration, without a reduction in cell motility (Fig. 2e and f). Together, these data indicate that mutant LRRK2 causes deficits in proper centrosome positioning with effects on polarity required for cells to properly respond to directional migration signals.

### Distinct pathogenic LRRK2 mutants cause centrosomal cohesion deficits in a kinase activity-dependent manner

**Fig. 3:**
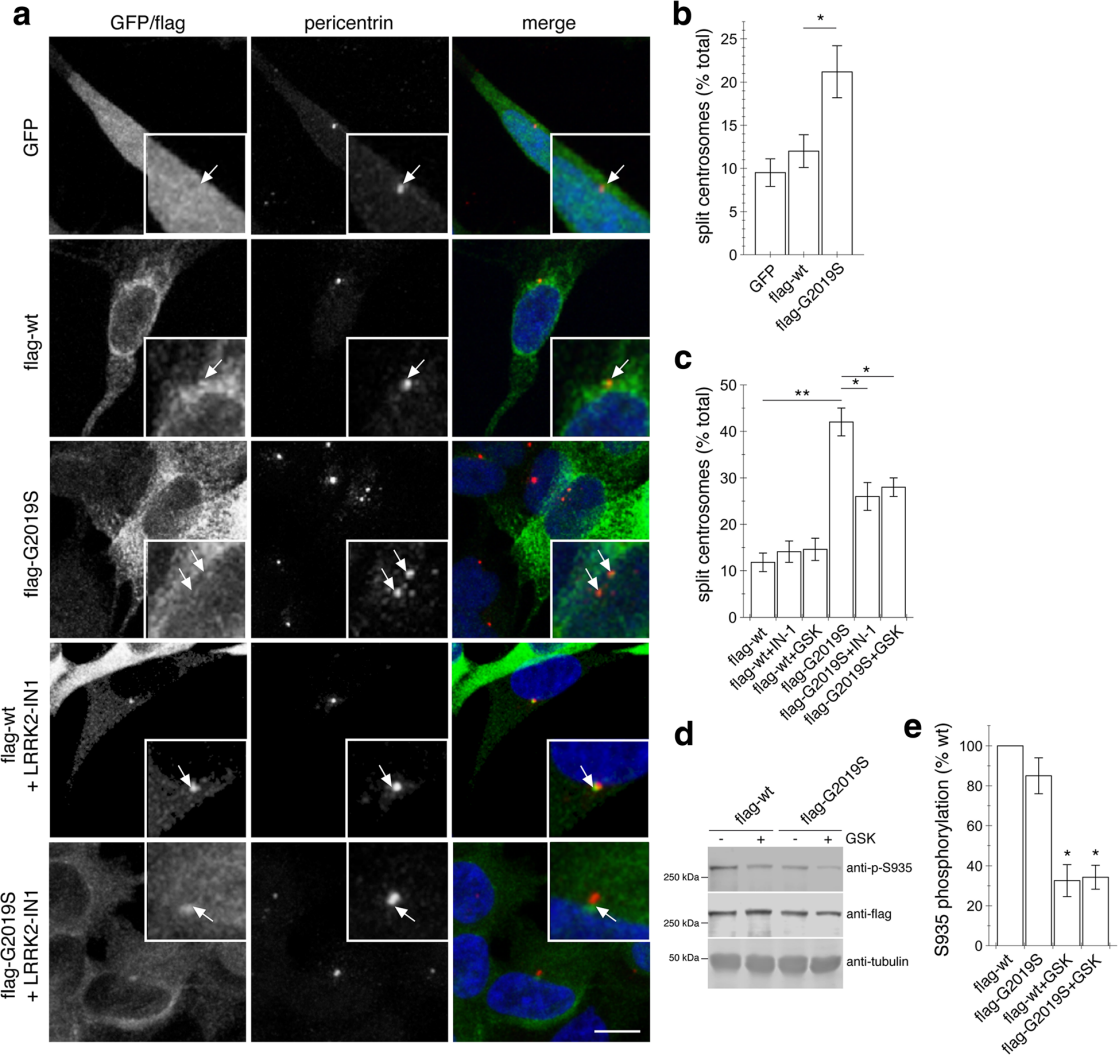
Pathogenic LRRK2 causes deficits in centrosome cohesion in SH-SY5Y cells. **a** Example of non-differentiated SH-SY5Y cells stably expressing GFP, or flag-tagged wildtype or G2019S-mutant LRRK2 as indicated, and stained for pericentrin and DAPI. Scale bar, 10 μm. **b** Quantification of the split centrosome phenotype in cells expressing GFP, or flag-tagged wildtype or G2019S-mutant LRRK2 as indicated. Around 20 cells with duplicated centrosomes were analyzed per condition. Bars represent mean ± s.e.m. (*n* = 4 independent experiments); *, *p* < 0.05. **c** Quantification of the split centrosome phenotype in cells expressing flag-tagged wildtype or G2019S-mutant LRRK2 as indicated, in either the absence or presence of kinase inhibitors (500 nM LRRK2-IN-1 or GSK2578215A for 1 h) as indicated. Around 20 cells with duplicated centrosomes were analyzed per condition. Bars represent mean ± s.e.m. (*n* = 3 independent experiments); **, *p* < 0.01; *, *p* < 0.05. **d** Cells were either left untreated or incubated with 500 nM GSK2578215A for 1 h, and extracts analyzed for phosphorylated (p-S935) or total (flag) LRRK2. **e** Quantification of S935 dephosphorylation in cells expressing flag-tagged wildtype or G2019S-mutant LRRK2 as indicated, in either the absence or presence of 500 nM GSK2578215A for 1 h. Bars represent mean ± s.e.m. (*n* = 3); *, *p* < 0.05

In dividing cells, centrosomes duplicate in S phase, but are held together by a flexible linker which gradually elongates during S and G2, allowing the duplicated centrosomes to mature by accumulating pericentriolar material. At the G2/M transition, the flexible linker holding the centrosomes together is lost, and mature centrosomes can subsequently constitute the poles of the mitotic spindle [26]. To determine for possible centrosomal alterations in dividing cells, we examined cells with duplicated centrosomes from non-differentiated SH-SY5Y cells. When quantifying cells where duplicated centrosomes could be clearly visualized as separate from each other, the mean distance between duplicated split centrosomes in GFP expressing cells was 1.49 ± 0.07 μm (mean ± s.e.m., *n* = 13 cells), and centrosomes were therefore scored as split when > 1.5 μm apart. As compared to GFP or wildtype LRRK2, pathogenic G2019S LRRK2 was found to significantly increase the percentage of cells with split centrosomes, indicative of a centrosomal cohesion deficit (Fig. 3a and b). To assess whether such centrosomal alterations induced by mutant LRRK2 were dependent on LRRK2 kinase activity, we evaluated the effects of two structurally distinct and specific LRRK2 kinase inhibitors, and quantified dephosphorylation of S935 on LRRK2 as an established readout for kinase inhibition [37, 38]. Short-term addition of these inhibitors significantly reverted the observed cohesion deficits in G2019S-LRRK2 expressing cells (Fig. 3c) and inhibited kinase activity as assessed by S935 dephosphorylation (Fig. 3d, e), suggesting that these cohesion deficits are kinase activity-mediated.

We next wondered whether the centrosomal cohesion deficits comprised a phenotype shared amongst distinct pathogenic LRRK2 mutants. For this purpose, HEK293T cells were transiently transfected with various mutant LRRK2 constructs. Similar to SH-SY5Y cells, the mean distance between duplicated split centrosomes in non-transfected HEK293T cells was 1.38 ± 0.05 μm (mean ± s.e.m., *n* = 12 cells), and centrosomes were scored as split when > 1.5 μm apart. As compared to non-transfected cells or to cells expressing wildtype LRRK2, a larger percentage of duplicated centrosomes displayed a split phenotype in cells expressing either G2019S-, R1441C- or Y1699C-mutant LRRK2, respectively, which was not observed with a kinase-dead G2019S-K1906 M mutant (Additional file 1: Figure S1a and b), even though all LRRK2 variants were expressed to similar degrees (Additional file 1: Figure S1c). As previously described [19], under the overexpression conditions in HEK293T cells employed here, pathogenic mutant LRRK2 also displayed more visible accumulation at and/or around centrosomes as compared to wildtype or kinase-inactive G2019S-K1906 M mutant LRRK2, which were cytosolic in the majority of cells (Additional file 1: Figure S1a and d), and additional localization of R1441C and Y1699C mutant LRRK2 to filamentous structures could be observed in some cells as well (Additional file 1: Figure S1e) [39]. Alterations in centrosomal cohesion were not due to aggregation-related events of LRRK2 protein overexpression, as triggering the formation of pericentrosomal protein aggregates (aggresomes) either by proteasomal inhibition in LRRK2-expressing cells, or by overexpression of a mutant version of huntingtin protein, did not cause centrosome splitting (Additional file 1: Figure S1f). Consistent with premature centrosome splitting in pathogenic LRRK2-expressing cells, the duplicated centrosomes were smaller than normal, mature centrosomes as determined in control or wildtype LRRK2-transfected cells (Additional file 1: Figure S1g and h). Thus, the observed centrosomal cohesion deficits seem to be a cellular feature shared amongst all three pathogenic LRRK2 mutants.

To assess whether premature centrosome splitting induced by mutant LRRK2 was dependent on LRRK2 kinase activity, we evaluated the effects of two distinct and selective LRRK2 kinase inhibitors [37, 38]. Addition of either inhibitor to wildtype LRRK2-expressing cells caused rapid recruitment of LRRK2 to the centrosome, but such centrosomal localization of kinase-inhibited LRRK2 did not cause centrosome splitting (Additional file 2: Figure S2a-c). However, whilst not causing a change in the centrosomal localization of pathogenic LRRK2 (Additional file 2: Figure S2d), treatment with either inhibitor reversed centrosome splitting in mutant LRRK2-expressing cells (Additional file 2: Figure S2e). Inhibitor-mediated reversal of centrosomal cohesion deficits were observed with all three pathogenic LRRK2 mutants (Additional file 2: Figure S2e,f), indicating that premature centrosome splitting is a shared feature of distinct pathogenic LRRK2 mutants and mediated by the kinase activity in both HEK293T and SH-SY5Y cells.

### Centrosomal cohesion deficits are detectable in two distinct peripheral cell types from LRRK2 PD patients as compared to healthy controls

**Fig. 4:**
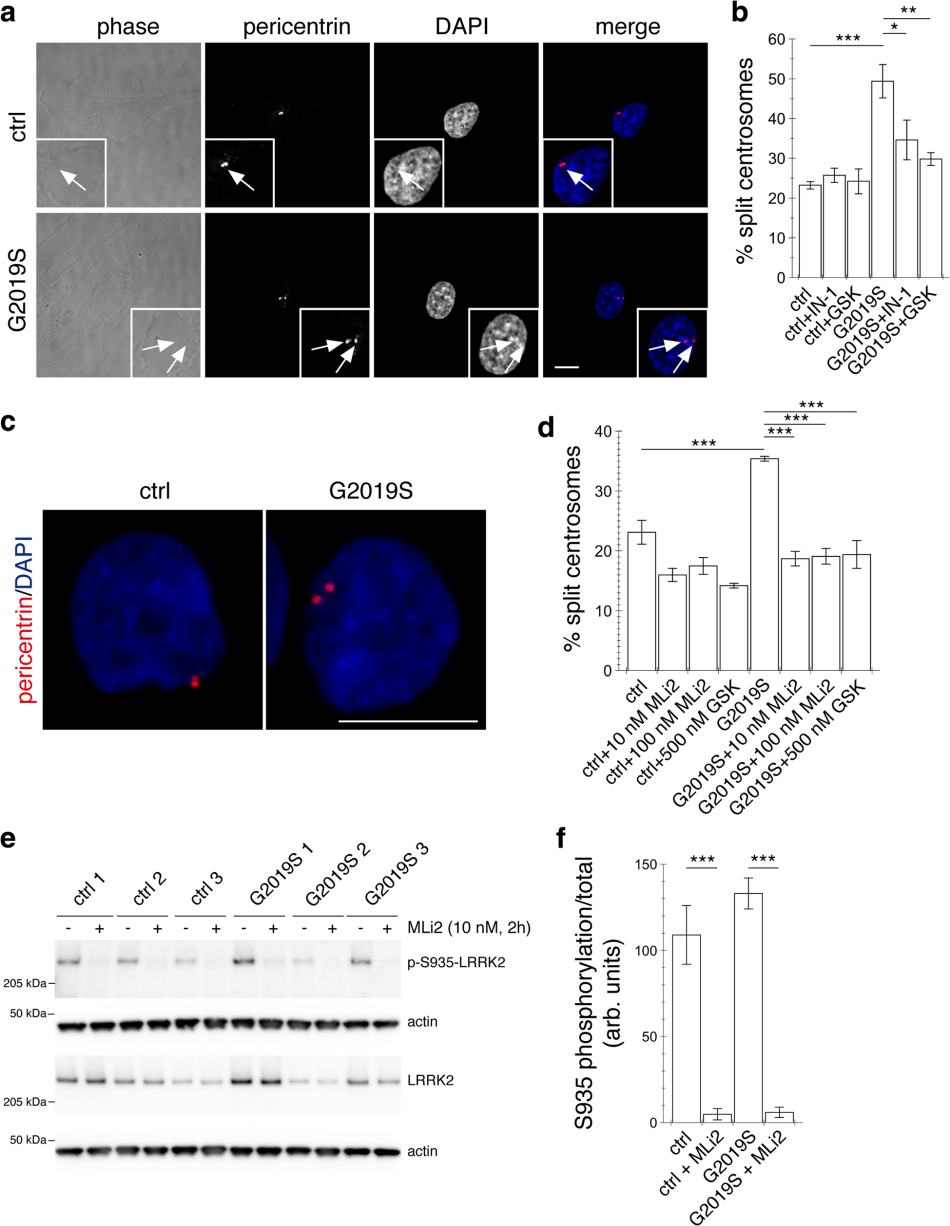
Centrosome splitting in human dermal fibroblasts and lymphoblasts from G2019S mutant LRRK2 PD patients compared to healthy controls. **a** Example of control (ctrl) and G2019S mutant LRRK2 PD patient fibroblast stained with pericentrin antibody and DAPI. Scale bar, 10 μm. **b** Centrosome phenotype was quantified from 300 cells per line, and from 5 control and 5 G2019S mutant LRRK2 fibroblast lines. Control or G2019S mutant LRRK2 fibroblasts were treated with LRRK2-IN-1 (500 nM) or GSK2578215A (500 nM) for 60 min. Bars represent mean ± s.e.m. (between five independent lines). ***, *p* < 0.005; **, *p* < 0.01; *, *p* < 0.05. **c** Example of control (ctrl) and G2019S mutant LRRK2 PD patient lymphoblast stained with pericentrin antibody and DAPI. Scale bar, 10 μm. **d** Centrosome phenotype was quantified from 200 to 300 cells per line, and from three control and three G2019S mutant LRRK2 lymphoblast lines. Control or G2019S mutant LRRK2 lymphoblasts were treated with MLi2 (10 nM or 100 nM) or GSK2578215A (500 nM) for 2 h. Bars represent mean ± s.e.m. (between three independent lines). ***, *p* < 0.005. **e** Cells were either left untreated or incubated with 10 nM MLi2 as indicated, and extracts analyzed for phosphorylated (p-S935) or total LRRK2. **f** Quantification of S935 dephosphorylation in either control or G2019S-mutant LRRK2 lymphoblasts as indicated, in either the absence or presence of 10 nM MLi2. Bars represent mean ± s.e.m. (*n* = 3 lines each); ***, *p* < 0.005

LRRK2 is known to be expressed in non-neuronal cells such as fibroblasts or lymphoblasts [40], and these patient-derived cells may comprise promising cellular models for future pharmacodynamic assays in clinical studies employing LRRK2 kinase inhibitors. Therefore, we next used either skin fibroblasts or lymphoblasts from age- and sex-matched G2019S mutant LRRK2 PD patients and healthy controls, respectively. Primary fibroblasts from five G2019S mutant LRRK2 PD patients displayed increased centrosome splitting as compared to five control patients, which was reverted by application of either GSK2578215A or LRRK2-IN-1 kinase inhibitors, respectively (Fig. 4a and b). Similarly, lymphoblasts from three G2019S mutant LRRK2 PD patients displayed deficits in centrosomal cohesion as compared to three healthy control cells. Such deficits were reverted by application of 500 nM GSK2578215A kinase inhibitor (Fig. 4c and d). In addition, application of either 10 nM or 100 nM of MLi2, a recently developed novel and highly selective LRRK2 kinase inhibitor [41] also reverted the centrosomal cohesion deficits (Fig. 4c and d), and inhibited LRRK2 kinase activity as assessed by S935 dephosphorylation (Fig. 4e and f). Together, these data indicate that endogenous mutant LRRK2 protein causes centrosomal alterations in a kinase activity-dependent manner also in two distinct patient-derived cell types.

### Pathogenic LRRK2-induced centrosomal cohesion deficits correlate with aberrant centrosomal accumulation of phosphorylated Rab8a

We next aimed to determine the mechanism(s) by which pathogenic LRRK2 may cause the observed centrosomal alterations. Recent studies have identified a subset of Rab GTPases as LRRK2 kinase substrates, with Rab8a being one of the most prominent substrates [3], and known to be involved in centrosome-related events. Indeed, we confirmed that Rab8a was subject to phosphorylation by LRRK2 in vitro, that phosphorylation was increased with pathogenic as compared to wildtype LRRK2, and that phosphorylation was largely abolished when mutating the previously identified phosphorylation site (T72) in the switch II domain (Additional file 3: Figure S3a and b). Moreover, we found that phosphorylation was not altered by either GDP or GTP binding to Rab8a (Additional file 3: Figure S3a-d), indicating that it was not dependent on the nucleotide-bound status of Rab8a.

Rab proteins interact with a variety of regulatory proteins. For example, the localization of Rab proteins is regulated by binding to GDP dissociation inhibitor (GDI1/2), which is able to deliver as well as extract Rab proteins from membranes [42], and their activity is modulated by binding to GDP/GTP exchange factors (GEFs) [43]. To test for differential interactions with regulatory proteins, we next generated phospho-deficient (Rab8a-T72A) and phospho-mimetic (Rab8a-T72D/Rab8a-T72E), as well as GTP-preferring (Rab8a-Q67L) and GDP-preferring (Rab8a-T22 N) Rab8a variants, which were all expressed to similar degrees (Additional file 3: Figure S3e). All mutants with the exception of Rab8a-T22 N were competent to bind GTP and GDP, and the rates of GTP/GDP dissociation were not affected by the mutations, indicating that mimicking phosphorylation does not change the nucleotide state of Rab8a (Additional file 3: Figure S3f-i). As previously described [3], whilst GDI1/2 coimmunoprecipitated with wildtype Rab8a as determined by mass spectroscopy, this interaction was lost with the phospho-mimetic Rab8a mutants (Additional file 4: Figure S4a-c), which also displayed a decreased interaction with the GEF Rabin8 (Rab3IP) (Additional file 4: Figure S4d and e), suggesting that mimicking Rab8a phosphorylation interferes with its interaction with multiple regulatory proteins. When expressed on their own, phospho-mimetic Rab8a mutants did not cause centrosomal cohesion deficits and were found to be largely cytosolic (Additional file 4: Figure S4f,g). Thus, these mutants cannot properly mimick a phosphorylated version of Rab8a in a cellular context.

**Fig. 5:**
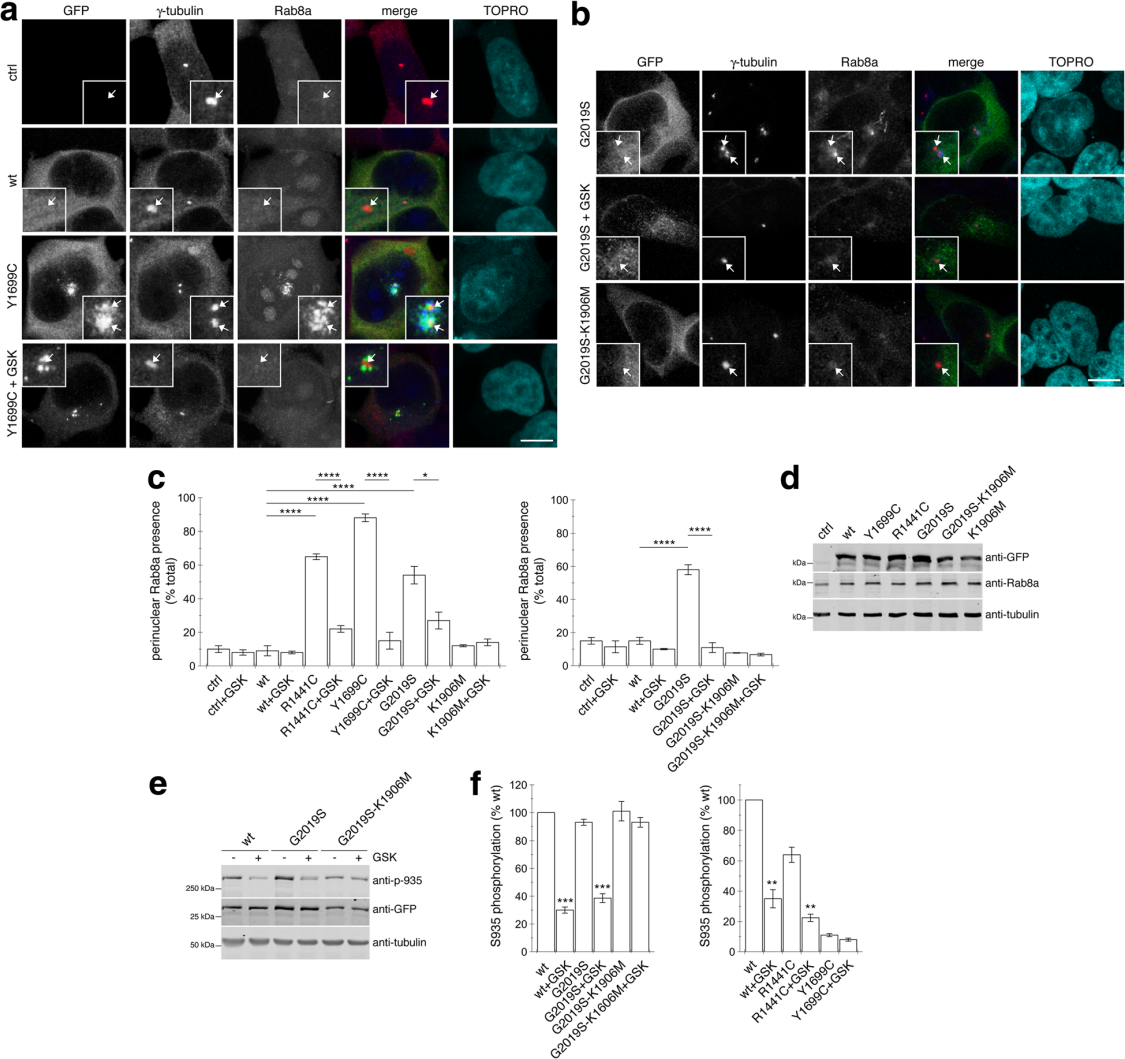
Pathogenic LRRK2 causes kinase-dependent pericentrosomal/centrosomal accumulation of endogenous Rab8a. **a**, **b** Examples of non-transfected HEK293T cells (ctrl) or cells transfected with either wildtype or pathogenic LRRK2, or with kinase-dead pathogenic LRRK2 as indicated, and stained with γ-tubulin antibody, Rab8a antibody (rabbit polyclonal Rab8a antibody for panel a, sheep polyclonal antibody for panel **b**), and TOPRO. Scale bar, 5 μm. **c** Quantification of the percentage of cells displaying pericentrosomal Rab8a staining in either non-transfected cells (ctrl), or pathogenic LRRK2-transfected cells as indicated, either in the absence or presence of GSK2578215A (GSK) (500 nM, 1 h). Bars represent mean ± s.e.m., (*n* = 3 independent experiments); ****, *p* < 0.001; *, *p* < 0.05. **d** Cells were transfected with the indicated constructs, and extracts blotted for GFP-tagged LRRK2, endogenous Rab8a, and tubulin as loading control. **e** Cells were either left untreated or incubated with 500 nM GSK2578215A for 1 h as indicated, and extracts analyzed for phosphorylated (p-S935) or total (GFP) LRRK2. **f** Quantification of S935 dephosphorylation in cells expressing wildtype or mutant LRRK2 as indicated, in either the absence or presence of 500 nM GSK2578215A for 1 h. Bars represent mean ± s.e.m. (*n* = 3); ***, *p* < 0.005; **, *p* < 0.01

Rab8a has been shown to be localized to a pericentrosomal recycling compartment which is in direct contact with the centrosome to regulate a variety of centrosome-related events [20–22]. We thus wondered whether pathogenic LRRK2 may alter the subcellular localization of endogenous Rab8a. Whilst endogenous Rab8a was rarely localized to a pericentrosomal/centrosomal compartment in control cells, all pathogenic LRRK2 mutants caused a prominent increase in the amount of cells displaying pericentrosomal/centrosomal Rab8a accumulation (Fig. 5a and b) without changes in the total levels of endogenous Rab8a protein (Fig. 5c).

Rab8a can also localize to the Golgi complex which is in tight contact with the centrosome [24, 44], and such centrosome-Golgi nexus is known to be important for cell polarity [45]. To determine whether the pericentrosomal/centrosomal accumulation of Rab8a is reflective of enhanced Golgi association, non-transfected or LRRK2-transfected cells were either treated with nocodazole, which causes Golgi fragmentation and dispersal, or with brefeldin A, which causes redistribution of the Golgi complex into the ER [46]. Golgi dispersal or complete Golgi redistribution did not alter the accumulation of Rab8a in pathogenic LRRK2-expressing cells (Additional file 5: Figure S5), indicating that Rab8a genuinely associates with a centrosomal/pericentrosomal compartment.

**Fig. 6:**
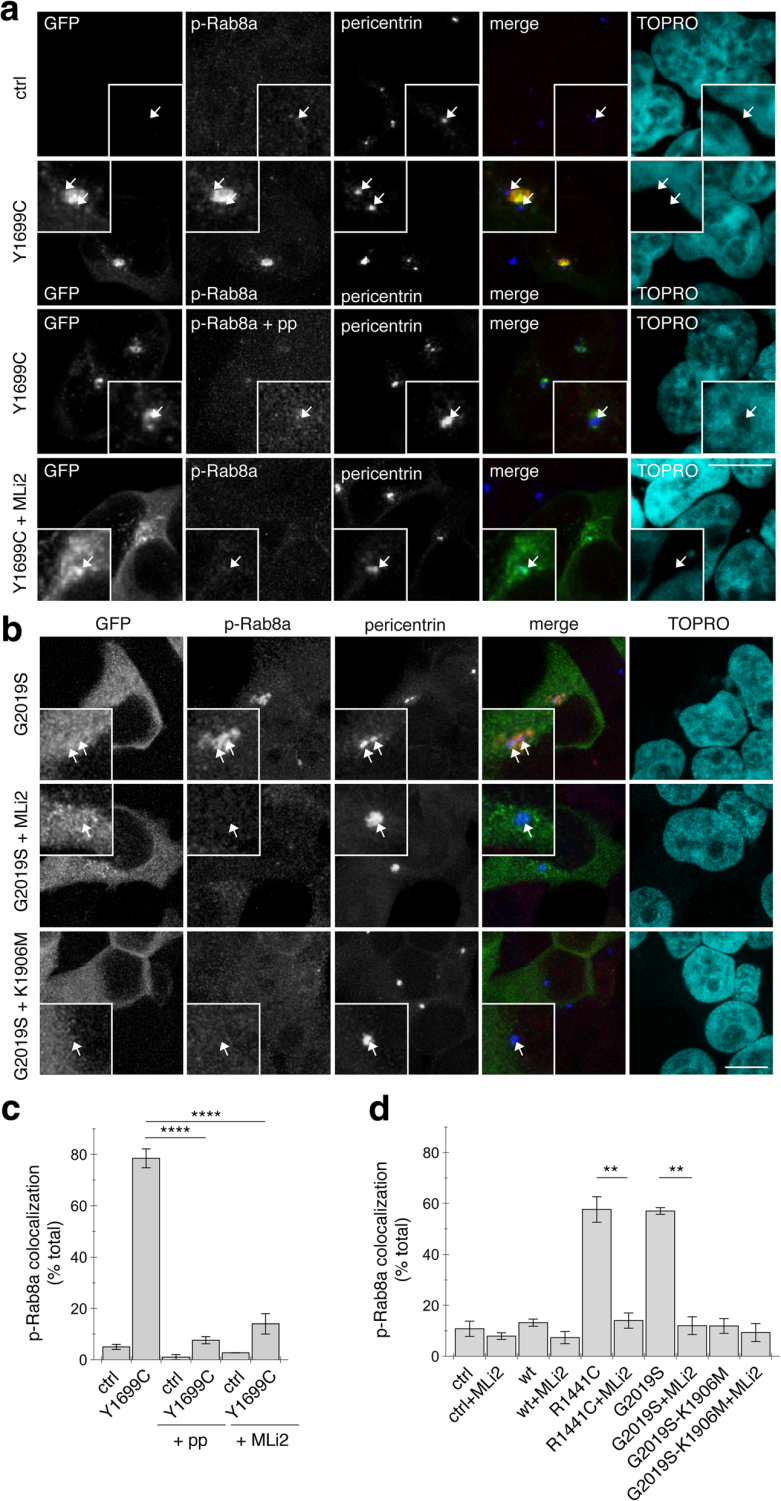
Pathogenic LRRK2 causes kinase-dependent pericentrosomal/centrosomal accumulation of endogenous phospho-Rab8a. **a**, **b** Cells were transfected with pathogenic LRRK2 or with kinase-dead pathogenic LRRK2 as indicated, and stained using an anti-phospho-T72-Rab8a antibody preabsorbed either with dephospho-peptide (p-Rab8a) or with phospho-peptide (p-Rab8a + pp), or with an anti-phospho-T72-Rab8a antibody preabsorbed with dephospho-peptide upon incubation of cells with 100 nM MLi2 for 60 min prior to immunocytochemistry as indicated. Scale bar, 5 μm. **c** Quantification of the percentage of non-transfected or transfected cells displaying phospho-Rab8a staining colocalizing with centrosomes within a 3 μm diameter circle in either the absence or presence of antibody preabsorption with peptides or pretreatment of cells with MLi2 as described above. Around 50 cells were quantified per condition per experiment. Bars represent mean ± s.e.m., (*n* = 3 independent experiments); ****, *p* < 0.001. **d** Quantification of the percentage of non-transfected or transfected cells displaying phospho-Rab8a staining colocalizing with centrosomes as described above. Around 50 cells were quantified per condition per experiment. Bars represent mean ± s.e.m., (*n* = 3 independent experiments); **, *p* < 0.01

The centrosomal/pericentrosomal Rab8a accumulation was reverted upon application of the LRRK2 kinase inhibitor GSK2578215A (Fig. 5a and b). Whilst S935 phosphorylation is a reliable readout to determine whether pharmacological kinase inhibitors block the LRRK2 kinase activity, it is not predictive of kinase activity of pathogenic mutants per se [47–53]. Indeed, whilst varying S935 phosphorylation levels were detected in wildtype and mutant LRRK2, application of GSK2578215A effectively inhibited the activity of both wildtype and mutant LRRK2 variants as measured by S935 dephosphorylation (Fig. 5d, e). Furthermore, Rab8a accumulation was not observed when expressing the kinase-dead pathogenic LRRK2 mutant G2019S-K1906 M (Fig. 5b). We thus wondered whether the accumulating Rab8a species may be a phosphorylated version of the protein. For this purpose, non-transfected or pathogenic LRRK2-transfected cells were stained with an antibody raised for the specific detection of phospho-T72-Rab8a [3]. Mutant LRRK2 expression caused a centrosomal/pericentrosomal accumulation of phosphorylated Rab8a, which was not observed when preincubating the phospho-antibody with phospho-peptide, and was reversed upon pretreatment of cells expressing either pathogenic G2019S, R1441C or Y1699C LRRK2 with MLi2 (Fig. 6a-c). Therefore, the accumulating Rab8a species in LRRK2-expressing HEK293T cells seems to represent a phosphorylated version of the protein.

Amongst all cell types analyzed here, lymphoblasts seem to contain the highest levels of endogenous Rab8a (Additional file 6: Figure S6a). We therefore attempted to determine whether alterations in endogenous phospho-Rab8a accumulation could be detected in lymphoblasts derived from G2019S LRRK2-PD patients as compared to healthy controls. No significant differences in the total levels of Rab8a were detected between lymphoblasts from control versus G2019S LRRK2-PD patients (Additional file 6: Figure S6b and c). Staining with the phospho-Rab8a antibody revealed a pericentrosomal/centrosomal accumulation of phospho-Rab8a in control and G2019S LRRK2-PD samples, which was absent when preincubating the antibody with phosphopeptide (Additional file 6: Figure S6d). Quantification of the intensity of the fluorescence signal revealed a slight, but not significant increase in phospho-Rab8a staining in G2019S LRRK2-PD samples as compared to controls (Additional file 6: Figure S6e), suggesting that higher affinity phospho-antibodies will be required to detect possible changes in the localization of endogenous phosphorylated Rab8a.

**Fig. 7:**
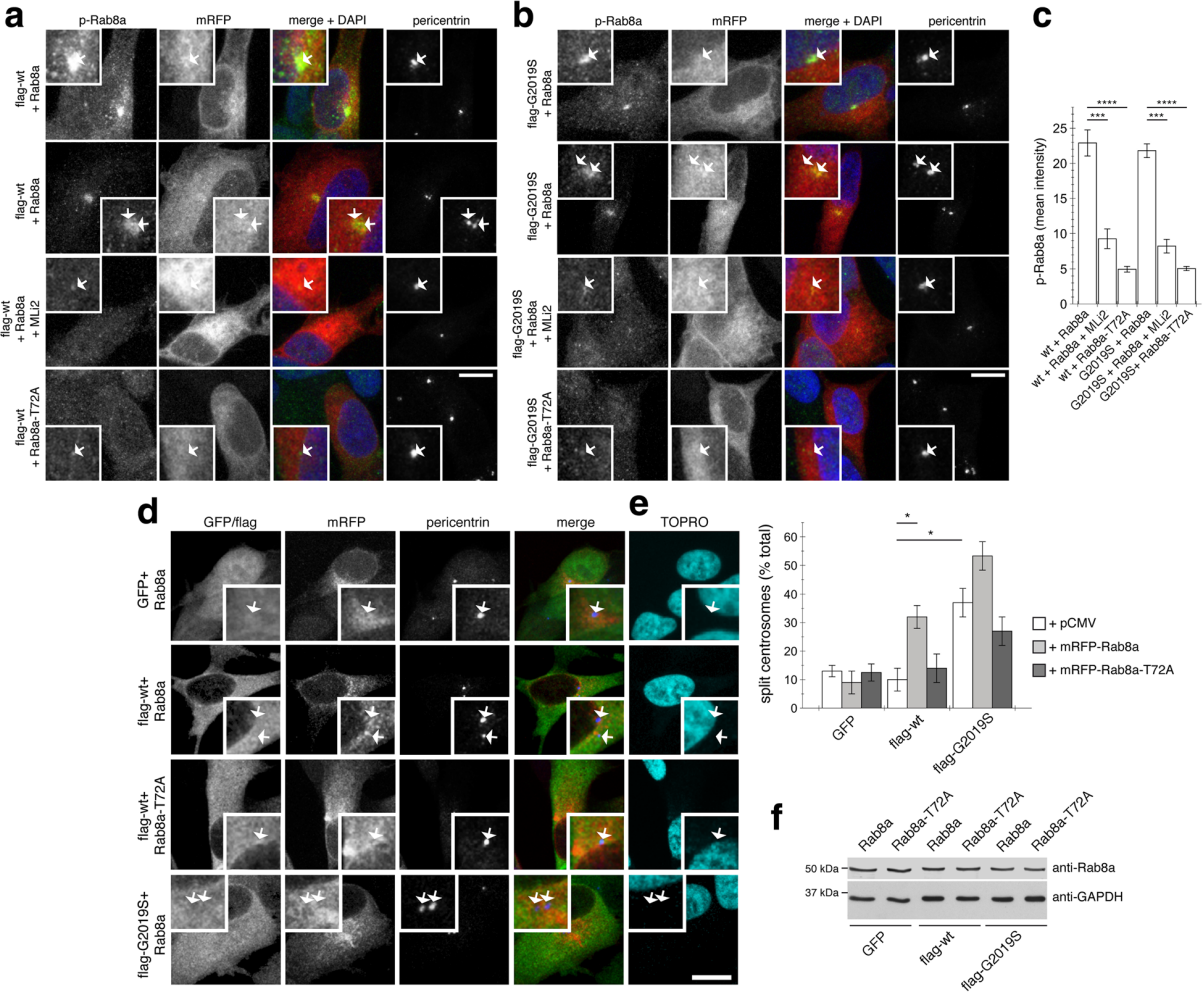
Expression of wildtype but not phosphorylation-deficient Rab8a causes centrosomal accumulation of phospho-Rab8a and centrosome cohesion deficits in wildtype LRRK2-expressing SH-SY5Y cells. **a** Example of SH-SY5Y cells stably expressing flag-tagged wildtype LRRK2, and transfected with mRFP-tagged wildtype or T72A-mutant Rab8a as indicated, stained with an anti-phospho-T72-Rab8a antibody preabsorbed with dephosphopeptide, for pericentrin and DAPI. Where indicated, cells were treated with 100 nM MLi2 for 2 h before immunocytochemistry. Note that phospho-Rab8 can be detected both in cells with one centrosome (first panel) as well as in cells with duplicated centrosomes (second panel). Scale bar, 10 μm. **b** Same as in **a**, but SH-SY5Y cells stably expressing flag-tagged G2019S mutant LRRK2. Scale bar, 10 μm. **c** Quantification of mean fluorescence intensity of phospho-Rab8a signal as described in Methods in cells either stably expressing wildtype (wt) or G2019S mutant LRRK2, transfected with RFP-tagged Rab8a or T72-mutant Rab8a, and treated with 100 nM MLi2 for 2 h before immunocytochemistry as indicated. Bars represent mean ± s.e.m., (*n* = 3 independent experiments); ****, *p* < 0.001; ***, *p* < 0.005. **d** Example of non-differentiated SH-SY5Y cells stably expressing GFP, flag-tagged wildtype or G2019S-mutant LRRK2 as indicated, and transfected with Rab8a or phosphorylation-deficient Rab8a (Rab8a-T72A) as indicated, and stained for pericentrin and TOPRO. Scare bar, 10 μm. **e** Quantification of the split centrosome phenotype in SH-SY5Y cells from the type of experiments depicted in **a**. Bars represent mean ± s.e.m. (*n* = 3 experiments); *, *p* < 0.05. **f** SH-SY5Y cells stably expressing GFP, flag-tagged wildtype or G2019S-mutant LRRK2 as indicated were transfected with mRFP-tagged wildtype or phosphorylation-deficient Rab8a (Rab8a-T72A) as indicated, and extracts blotted for Rab8a levels and GAPDH as loading control

The phospho-Rab8a antibody was also not able to detect differences in the centrosomal accumulation of endogenous phospho-Rab8a in wildtype versus G2019S LRRK2-mutant SH-SY5Y cells (Fig. 7a and b). Therefore, as a means to increase phosphorylated Rab8a species, we expressed wildtype Rab8a in either wildtype or G2019S LRRK2-mutant SH-SY5Y cells. Under these conditions, a drastic increase in Rab8a phospho-signal was observed (Fig. 7c and d), which was abolished when pretreating cells with a LRRK2 kinase inhibitor, and which was not observed when expressing the non-phosphorylatable Rab8a-T72A mutant (Fig. 7b-d), indicating that it was specifically detecting a LRRK2-phosphorylated version of Rab8a. Even though the expressed RFP-tagged Rab8a protein was widely distributed, the phospho-Rab8a signal was confined to a localization overlapping with that of a centrosomal marker, indicating that the phosphorylated Rab8a species preferentially accumulates in a centrosomal compartment (Fig. 7c and d). Moreover, phosphorylated Rab8a accumulated in a centrosomal compartment irrespective of whether the cells had duplicated centrosomes or not (Fig. 7c and d), suggesting that such localization reflects a general feature not limited to a specific phase of the cell cycle.

We next analyzed whether an increase in centrosomal phospho-Rab8a may cause the observed alterations in centrosomal cohesion. Expression of wildtype Rab8a in wildtype or G2019S mutant LRRK2-expressing SH-SY5Y cells caused a prominent deficit in centrosomal cohesion (Fig. 7e and f). Such cohesion deficits were not observed when expressing the non-phosphorylatable Rab8a-T72A mutant (Fig. 7e and f), even though both Rab8a variants were expressed to similar degrees (Fig. 7g). Premature centrosome splitting induced by G2019S-mutant LRRK2 was not further modulated by the non-phosphorylatable mutant (Fig. 7f), indicating that this mutant does not act as a dominant-negative in this context.

Similar results were obtained in HEK293T cells (Additional file 7: Figure S7). Co-expression of wildtype LRRK2 with wildtype Rab8a resulted in LRRK2-mediated Rab8a phosphorylation, which was further enhanced when co-expressing the distinct pathogenic, but not kinase-dead K1906 M or G2019S-K1906 M LRRK2 mutants (Additional file 7: Figure S7a). Co-expression of wildtype LRRK2 with wildtype Rab8a caused a centrosomal cohesion deficit which was not observed when expressing the non-phosphorylatable Rab8a-T72A mutant (Additional file 7: Figure S7b and c) or when treating cells with kinase inhibitor (Additional file 7: Figure S7d). Altogether, these data indicate that increasing the centrosomal amount of LRRK2-phosphorylated Rab8a correlates with the observed centrosomal cohesion deficits.

### Pathogenic LRRK2-induced centrosomal cohesion and polarity deficits are mediated by Rab8a

**Fig. 8:**
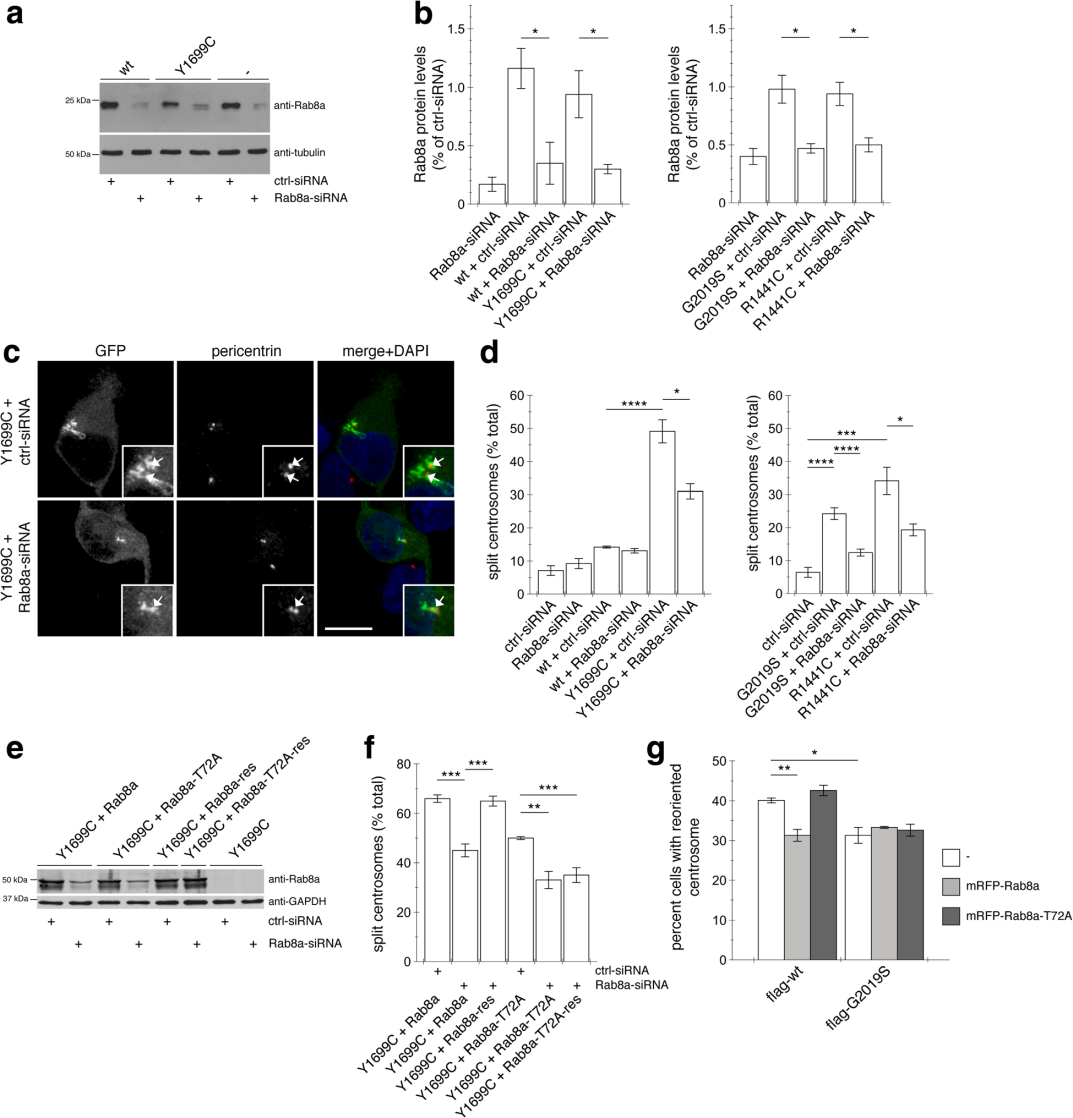
Knockdown of Rab8a significantly reverses the centrosomal deficits mediated by pathogenic LRRK2. **a** Representative Western blot of extracts from control cells (ctrl), or cells transfected with wildtype (wt) or Y1699C-mutant LRRK2, along with either ctrl-siRNA or Rab8a-siRNA as indicated, and blotted against Rab8a or tubulin as loading control. **b** Quantification of the type of experiments depicted in **a**, with levels of Rab8a normalized to tubulin and to Rab8a levels in the presence of ctrl-siRNA. Bars represent mean ± s.e.m. (*n* = 3–5 independent experiments); * *p* < 0.05. **c** Example of cells co-transfected with GFP-tagged pathogenic LRRK2 and either ctrl-siRNA or Rab8a-siRNA as indicated, and stained with pericentrin antibody and DAPI. Scale bar, 10 μm. **d** Quantification of the split centrosome phenotype in control cells transfected with either ctrl-siRNA or Rab8a-siRNA, or in cells co-transfected with wildtype or mutant LRRK2 as indicated. Bars represent mean ± s.e.m. (*n* = 3–5 independent experiments); **** *p* < 0.001; * *p* < 0.05. **e** Western blot of cell extracts transfected with either ctrl-siRNA or Rab8a-siRNA as indicated, and four hours later cotransfected with mutant LRRK2 and mRFP-tagged Rab8a, Rab8a-T72A, or siRNA-resistant versions thereof (res), and blotted against Rab8a and GAPDH as loading control. **f** Quantification of the split centrosome phenotype in the presence of ctrl-siRNA or Rab8a-siRNA, and in the presence of pathogenic mutant LRRK2 and mRFP-tagged Rab8a, Rab8a-T72A or siRNA-resistant versions thereof (res) as indicated. Bars represent mean ± s.e.m. (*n* = 3 independent experiments); *** *p* < 0.005; ** *p* < 0.01. **g** Quantification of centrosome reorientation in cells stably expressing flag-tagged wildtype or G2019S-mutant LRRK2 together with RFP-tagged wildtype or phospho-deficient T72A Rab8a 4 h after generating the wound (*t* = 4 h). Random orientation is expected to be 25%. *n* > 50 cells were quantified for each condition in each experiment. Bars represent mean ± s.e.m. (*n* = 3 independent experiments); **, *p* < 0.01; *, *p* < 0.05

To determine whether the centrosomal deficits caused by pathogenic LRRK2 kinase were Rab8a-mediated, HEK293T cells were transiently transfected with small interfering RNAs (siRNA) directed against a control sequence or against Rab8a. Cells transfected with the Rab8a-specific siRNA showed a significant decrease in Rab8a protein content when compared to control siRNA (Fig. 8a and b). Whilst knocking down Rab8a protein levels did not cause alterations in centrosomal cohesion in non-transfected cells or in cells transfected with wildtype LRRK2, it caused a significant reversal in the centrosomal cohesion deficits induced by pathogenic G2019S, R1441C or Y1699C LRRK2 expression (Fig. 8c and d).

As an additional means to assure that the observed reversal of the LRRK2-mediated centrosomal cohesion deficits were due to knockdown of Rab8a, we generated siRNA-resistant versions of Rab8a as well as Rab8a-T72A (Fig. 8e). Whilst siRNA of Rab8a reduced the mutant LRRK2-mediated centrosomal cohesion deficits in transiently transfected HEK293T cells, those deficits were restored in the presence of siRNA-resistant Rab8a, but not of siRNA-resistant Rab8a-T72A mutant (Fig. 8f). Therefore, Rab8a, and specifically a phosphorylatable form of Rab8a, is important for pathogenic LRRK2 to cause the observed centrosomal deficits.

Finally, when analyzing cell polarization upon scratch wounding, expression of wildtype Rab8a, but not phosphorylation-deficient Rab8a, caused a pronounced decrease in the percentage of cells with reoriented centrosomes in wildtype LRRK2-expressing cells after 4 h of wounding, whilst not further altering the deficits observed in G2019S LRRK2-expressing cells (Fig. 8g). Thus, the effects of pathogenic LRRK2 on centrosome positioning in the context of cell polarity also seem to involve a Rab8a-mediated phosphorylation event.

## Discussion

Here, we provide evidence that mutant LRRK2 causes centrosomal alterations in both dividing and non-dividing cells. Centrosomal deficits were observed with distinct pathogenic LRRK2 mutants and in distinct cell lines, as well as in two different patient-derived cell types. The effects were reverted by various specific and structurally distinct LRRK2 kinase inhibitors, suggesting that LRRK2 causes centrosomal alterations in a kinase activity-dependent manner. Together, the data indicate that endogenous pathogenic LRRK2 protein impacts upon the same cellular pathway, and highlight the potential applicability of such cellular readout as possible pharmacodynamic assay in clinical studies employing LRRK2 kinase inhibitors.

Our data confirm that Rab8a is robustly phosphorylated on T72 by LRRK2 in vitro, and further indicate that phosphorylation is largely independent on nucleotide-bound status. As compared to wildtype or phosphorylation-deficient Rab8a, phospho-mimetic Rab8a mutants did not display altered nucleotide binding/dissociation, but were unable to interact with GDI1/2 or with Rabin8. However, when transiently expressed in cells, the phospho-mimetic Rab8a mutants were not able to mimick the localization or action of phosphorylated Rab8a in intact cells. Both the negative charge and the size of the ionic shell produced by aspartate or glutamate substitutions are different from those of a phosphorylated residue at physiological pH. In addition, if the phosphorylation site serves as a recognition signal for a binding partner, phospho-mimetic mutants cannot bind as not fitting into the binding pocket [54, 55]. Therefore, and as observed here, phospho-mimetic mutations often fail to reproduce the changes to a protein caused by its phosphorylation.

Whilst clearly a LRRK2 kinase substrate, previous studies have shown that the stoichiometry of Rab8a phosphorylation in intact cells is low, with the phospho-state-specific Rab8a antibody only detecting phosphorylated protein by Western blotting techniques upon co-transfection with exogenous Rab8a [3]. By immunocytochemistry, we could detect accumulation of endogenous phosphorylated Rab8a in HEK293T cells expressing high levels of exogenous pathogenic LRRK2, or in SH-SY5Y cells stably expressing LRRK2 upon co-transfection with exogenous Rab8a. In lymphoblasts which express both high levels of endogenous LRRK2 and of Rab8a, a pericentrosomal/centrosomal phospho-Rab8a signal was detectable, but there were no statistically significant differences between G2019S LRRK2 mutant and control lymphoblasts. These data are consistent with the interpretation that the centrosomal effects mediated by pathogenic LRRK2 are, at least in part, mediated by Rab8a and Rab8a phosphorylation, even though currently available tools do not allow us to detect endogenous phospho-Rab8a in G2019S mutant lymphoblasts.

The LRRK2-mediated phosphorylation of Rab8a does not seem to cause a mere loss-of-function phenotype, as knockdown of Rab8a in control cells did not cause centrosomal cohesion deficits. Conversely, pathogenic LRRK2 expression caused centrosomal defects accompanied by an accumulation of endogenous phosphorylated Rab8a in a pericentrosomal/centrosomal compartment, and increasing the amount of phosphorylated Rab8a by coexpression of wildtype LRRK2 with wildtype but not phospho-deficient Rab8a caused centrosomal cohesion deficits and centrosomal polarity defects in a kinase activity-mediated manner as well. Finally, RNAi of Rab8a in pathogenic LRRK2-expressing cells caused a significant reversal of centrosomal cohesion deficits. Whilst future studies will be required to address whether the centrosomal cohesion deficits remaining upon Rab8a knockdown are mediated by remnant Rab8a, by other functionally redundant Rab protein LRRK2 kinase substrates such as Rab8b or Rab10 [3], or indeed by other, non-Rab-related LRRK2 substrates, these results indicate that a significant part of the phenotype is dependent on the presence of Rab8a. Altogether, our data are consistent with a model whereby pathogenic LRRK2 kinase activity causes an abnormal accumulation of phosphorylated Rab8a in a pericentrosomal/centrosomal compartment with various downstream effects on centrosome functioning as described here.

Centrosomal deficits were also observed in non-dividing cells. Differentiated SH-SY5Y cells expressing pathogenic LRRK2 displayed a deficit in cell polarity as evidenced by a significant increase in the amount of cells with abnormal positioning of the centrosome with respect to the longest neurite and a decrease in overall differentiation capability, consistent with previous reports that pathogenic LRRK2 interferes with neurite outgrowth [4, 6, 11, 15]. Mutant LRRK2 also caused deficits in cell polarity associated with an impairment in directional cell migration. Whilst both positive and negative effects of mutant LRRK2 on cell migration have been previously described [12–14], this may relate to cell type-specific differences in the position of the centrosome with respect to the leading edge of migratory cells [56]. In addition, impaired adult neurogenesis in mutant LRRK2-expressing cells seems to be accompanied by a reduction in the number of newly generated neurons migrating to the olfactory bulb [15]. As deficits in these processes may contribute to early clinical signs of PD such as anosmia, it will be interesting to determine whether these migrational deficits are due to a lack of proper centrosome positioning and cell polarization. Since Rab8a has also been implicated in neurite formation and polarized membrane transport [57, 58], and since polarity deficits were also observed when co-expressing wildtype but not phospho-deficient Rab8a with wildtype LRRK2, it is tempting to speculate that the effects on neurite outgrowth and directional migration may also involve altered Rab8a-mediated processes. Apart from its abnormal phosphorylation by LRRK2 [3], Rab8a has also been shown to be phosphorylated by a PINK1-mediated mechanism [59], and mutations in PINK1 are known to cause autosomal-recessive PD. In addition, Rab8a has been shown to modulate α-synuclein-mediated aggregation and toxicity in cellular and animal models of PD [60]. Thus, whilst the precise mechanism(s) remain to be further determined, there is increasing evidence linking abnormal Rab8a function with PD pathogenesis.

The link between centrosomal alterations as described here and its relevance to PD remains unclear. Deficits in adult neurogenesis have been reported to contribute to the age-dependent non-motor symptoms of PD patients [17, 18], and it will be interesting to determine whether alterations in centrosomal cohesion parallel the deficits in cell growth of neuronal precursor cells derived from LRRK2 PD patients in vitro, or the impairment of adult neurogenesis in mutant LRRK2-transgenic mice in vivo [15, 16]. In addition, as centrosomal alterations are frequently associated with cancer, the changes reported here may further contribute to the reported increased cancer risk in LRRK2 PD patients [61]. Importantly, centrosomes are the major microtubule-nucleating centers within a cell, and proper centrosome functioning and orientation ensure appropriate microtubule-mediated vesicular trafficking. Interestingly, pathogenic LRRK2 has been linked to alterations in microtubule stability [62] and to intracellular vesicular trafficking steps including the autophagy, endolysosomal and retromer-mediated trafficking pathways [4–9, 19, 63]. Therefore, the centrosomal alterations described here may contribute to the observed alterations in microtubule-mediated membrane trafficking pathways which have been directly related to the pathobiology of PD [64, 65].

In summary, we here provide evidence that pathogenic LRRK2 causes deficits in centrosome positioning and cohesion in a manner dependent on kinase activity and Rab8a phosphorylation. Our findings hopefully provide a useful framework for future studies aimed at determining the relative contribution of centrosomal deficits to the various cellular alterations described to be relevant in the context of PD pathogenesis and amenable to LRRK2 kinase inhibitor-mediated strategies.

## Conclusions

Our data indicate that pathogenic LRRK2 causes centrosomal polarity and cohesion deficits. Such centrosomal defects have impacts on neurite outgrowth, cell polarization and migration. These defects are LRRK2 kinase activity-mediated, and are also observed in patient-derived cells. The centrosomal deficits in both dividing and non-dividing cells are largely due to LRRK2-mediated Rab8a phosphorylation. Thus, our study reveals a novel role for pathogenic LRRK2 related to proper centrosome functioning, which may not only contribute to neurodegeneration, but may also account for some early manifestations of the disease and the increased incidence of cancer seen in PD patients.

## Additional files

Additional file 1: Figure S1. Distinct pathogenic LRRK2 mutants cause deficits in centrosome cohesion in transfected HEK293T cells. (DOCX 1160 kb)

Additional file 2: Figure S2. Pathogenic LRRK2 disturbs centrosome cohesion in a kinase-dependent manner. (DOCX 1155 kb)

Additional file 3: Figure S3. LRRK2 phosphorylates Rab8a at T72, and phosphomimetic mutants do not display altered nucleotide binding or retention. (DOCX 1005 kb)

Additional file 4: Figure S4. Differential interactions of wildtype and phospho-mimetic Rab8a mutants with GDI1/2 and Rabin8, effects on centrosome splitting and subcellular localization. (DOCX 824 kb)

Additional file 5: Figure S5. Golgi dispersal/disruption has no effect on LRRK2-mediated pericentrosomal/centrosomal accumulation of Rab8a. (DOCX 1670 kb)

Additional file 6: Figure S6. Rab8a protein levels and pericentrosomal/centrosomal accumulation of phosphorylated Rab8a in lymphoblasts from control and G2019S mutant LRRK2 PD patients. (DOCX 636 kb)

Additional file 7: Figure S7. Detection of phospho-Rab8a in pathogenic LRRK2-expressing cells as well as in cells co-transfected with wildtype LRRK2 and wildtype Rab8a, but not phospho-deficient Rab8a. (DOCX 958 kb)

## Abbreviations

GDI: GDP dissociation inhibitor
GEFs: GDP/GTP exchange factors
LRRK2: Leucine-rich repeat kinase 2
PD: Parkinson’s disease
siRNA: Small interfering RNAs
