# Supplementary material for: Parkinson disease-associated mutations in LRRK2 cause centrosomal defects via Rab8a phosphorylation
Source: Mol Neurodegener. 2018 Jan 23;13:3. doi: 10.1186/s13024-018-0235-y (PMC5778812; doi:10.1186/s13024-018-0235-y)
Supplement: Supplementary file 1 — Distinct pathogenic LRRK2 mutants cause deficits in centrosome cohesion in transfected HEK293T cells. (DOCX 1160 kb) [file 13024_2018_235_MOESM1_ESM.docx]

**
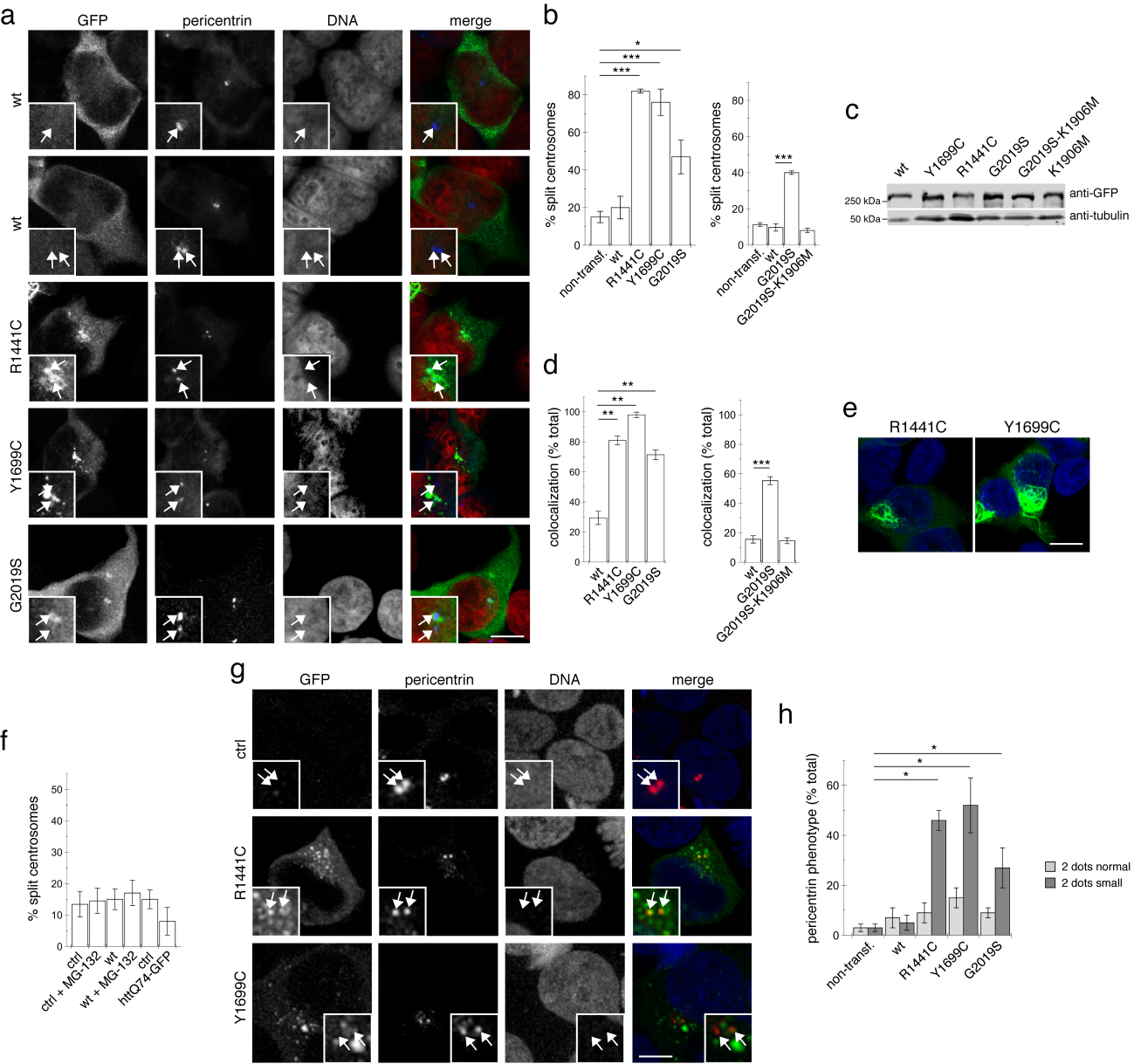
**

**Additional file 1: Figure S1.** Distinct pathogenic LRRK2 mutants cause deficits in centrosome cohesion in transfected HEK293T cells. **a** Examples of transfected cells displaying either one centrosome (interphase), or upon duplication and concomitant maturation (G2), two attached centrosomes (first two panels in wildtype LRRK2-expressing cells). Mutant LRRK2-expressing cells display a split centrosome phenotype. Scale bar, 5 μm. **b** The split centrosome phenotype was quantified by determining the percentage of cells displaying two pericentrin-positive structures with a distance between their centers > 1.5 μm, as compared to two attached (≤ 1.5 μm) structures. Around 30 cells with duplicated centrosomes were analyzed per condition per experiment. Bars represent mean ± s.e.m. (n=3 independent experiments); ***, p < 0.005; *, p < 0.05. **c** Cells were transfected with constructs as indicated, and 40 μg of cellular lysates subjected to Western blotting with an anti-GFP antibody. Tubulin was used as loading control. **d** Quantification of percentage of cells displaying colocalization of GFP-tagged LRRK2 with centrosomes. Around 30 cells were quantified per experimental condition. Bars represent mean ± s.e.m. (n=3 independent experiments); ***, p < 0.005; **, p < 0.01. **e** Examples of filament-like localization of GFP-tagged R1441C and Y1699C mutant LRRK2. Scale bar, 10 μm. **f** Centrosome splitting was quantified from cells transfected with pCMV (ctrl) or wildtype LRRK2 and treated 42 h after transfection with 5 μM MG-132 for 6 h, or from cells transfected with pCMV (ctrl) or HttQ74-GFP 24 h after transfection. N=3 independent experiments. **g** For determination of centrosomal size, cells were fixed and processed as described in Methods. Note that colocalization of mutant LRRK2 with split centrosomes can be more or less extensive (arrows). Scale bar, 5 μm. **h** Quantification of centrosome size from experiments of the type depicted in A. An average of 30 cells with two centrosomes were analyzed for each condition. Bars represent mean ± s.e.m. (n=3 independent experiments); *, p < 0.05.
