# Supplementary material for: Parkinson disease-associated mutations in LRRK2 cause centrosomal defects via Rab8a phosphorylation
Source: Mol Neurodegener. 2018 Jan 23;13:3. doi: 10.1186/s13024-018-0235-y (PMC5778812; doi:10.1186/s13024-018-0235-y)
Supplement: Supplementary file 2 — Pathogenic LRRK2 disturbs centrosome cohesion in a kinase-dependent manner. (DOCX 1155 kb) [file 13024_2018_235_MOESM2_ESM.docx]

**
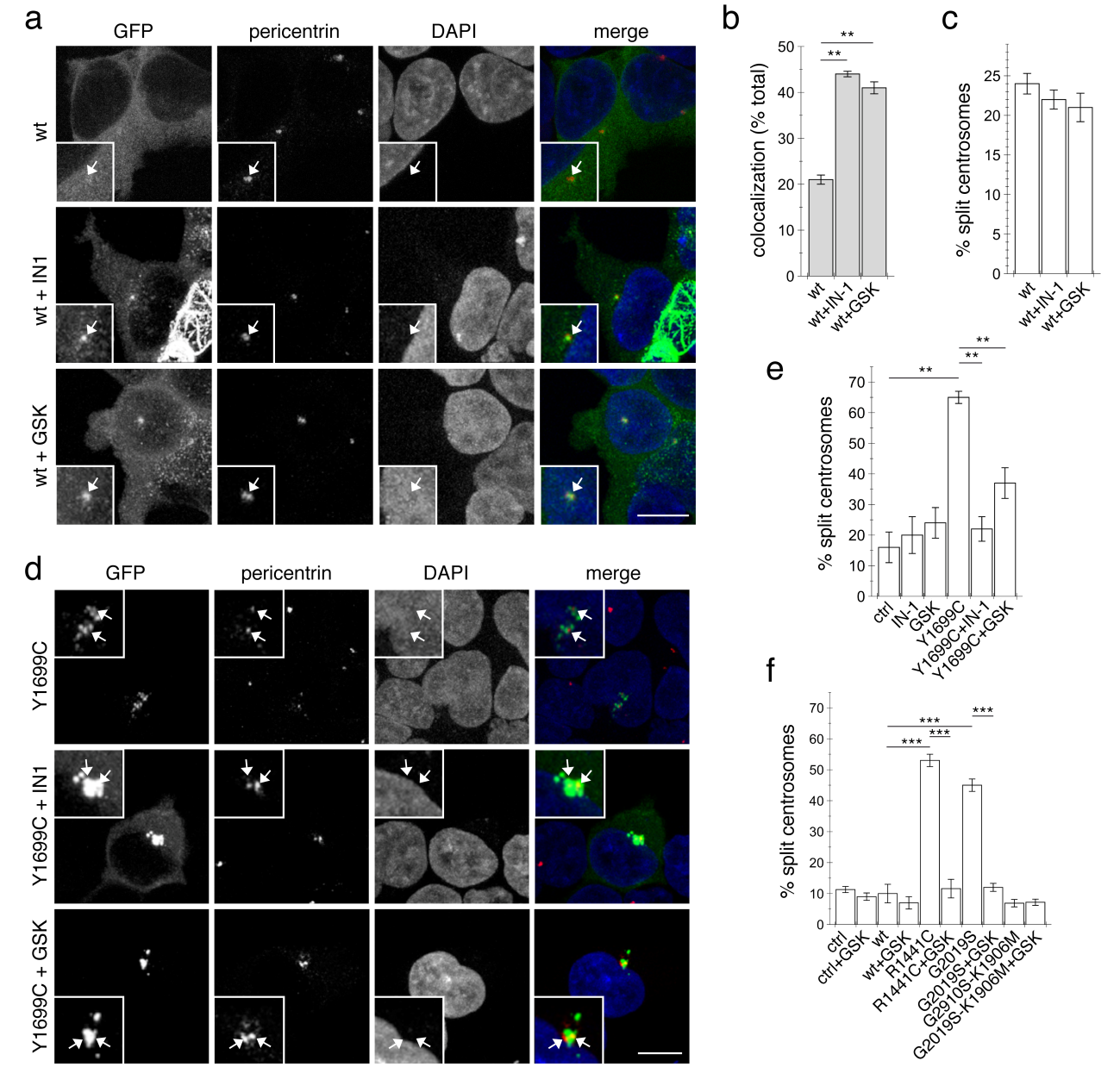
**

**Additional file 2: Figure S2.** Pathogenic LRRK2 disturbs centrosome cohesion in a kinase-dependent manner. **a** GFP-tagged wildtype LRRK2-transfected HEK293T cells were either untreated (ctrl), or treated with LRRK2-IN-1 (500 nM) or GSK2578215A (500 nM) for 60 min, followed by staining with pericentrin antibody. Scale bar, 10 μm. **b** Quantification of the percentage of LRRK2-expressing cells displaying visible colocalization with pericentrin in the absence or presence of kinase inhibitors as indicated. Around 100 cells were analyzed per condition. Bars represent mean ± s.e.m. (n=3 independent experiments); **, p < 0.01. **c** Quantification of the percentage of wildtype LRRK2-expressing cells displaying a split centrosome phenotype in the absence or presence of kinase inhibitors as indicated. Around 30 cells with duplicated centrosomes were analyzed per condition. Bars represent mean ± s.e.m. (n=3 independent experiments). **d** Examples of GFP-tagged Y1699C mutant LRRK2 in the absence (ctrl) or presence of LRRK2-IN-1 or GSK2578215A treatment as indicated above, followed by staining with pericentrin antibody. Scale bar, 10 μm. **e** The split centrosome phenotype was quantified as indicated above. An average of 30 cells with two centrosomes were analyzed for each condition. Bars represent mean ± s.e.m. (n=3 independent experiments); **, p < 0.01. **f** The split centrosome phenotype was quantified as indiated above and an average of 30 cells with two centrosomes analyzed for each condition. Bars represent mean ± s.e.m. (n=3 independent experiments); ***, p < 0.005.
