# Supplementary material for: Parkinson disease-associated mutations in LRRK2 cause centrosomal defects via Rab8a phosphorylation
Source: Mol Neurodegener. 2018 Jan 23;13:3. doi: 10.1186/s13024-018-0235-y (PMC5778812; doi:10.1186/s13024-018-0235-y)
Supplement: Supplementary file 3 — LRRK2 phosphorylates Rab8a at T72, and phosphomimetic mutants do not display altered nucleotide binding or retention. (DOCX 1005 kb) [file 13024_2018_235_MOESM3_ESM.docx]

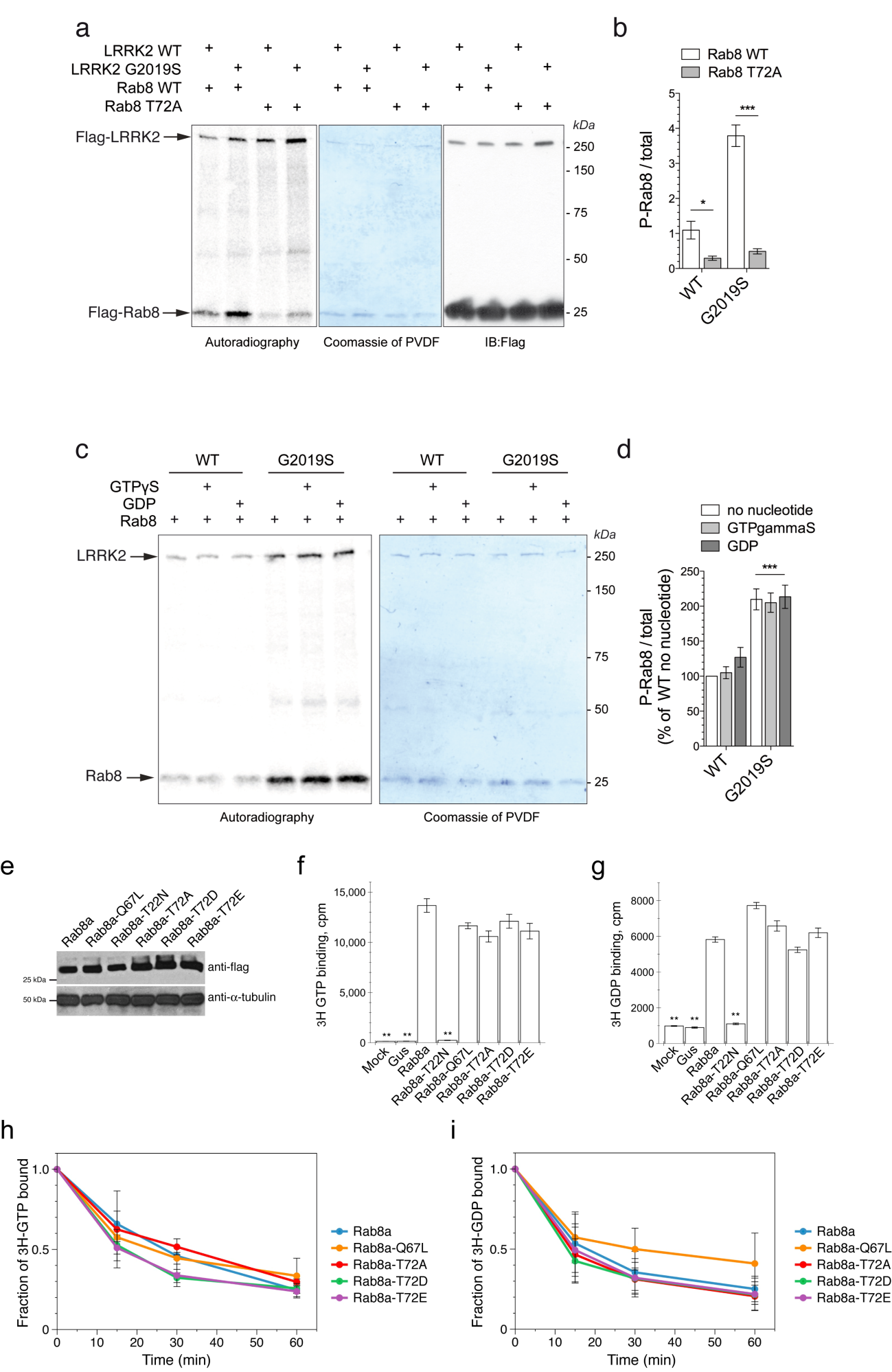


**Additional file 3: Figure S3.** LRRK2 phosphorylates Rab8a at T72, and phosphomimetic mutants do not display altered nucleotide binding or retention. **a** *In vitro* kinase assays using purified wildtype or G2019S mutant flag-tagged LRRK2 and purified wildtype or T72A mutant flag-tagged Rab8a (1:50 ratio). ^33^P incorporation was revealed by autoradiography (left panel), and total protein loading by Coomassie Brilliant Blue staining (middle panel) or anti-flag immunoblotting (right panel) of the same PVDF membrane. **b** Quantification of ^33^P incorporated into Rab8a as compared to total Rab8a as determined by Coomassie Brilliant Blue staining. Bars represent mean ± s.e.m. (n=3 independent experiments); *, p < 0.05; ***, p < 0.001 (two-way ANOVA with Bonferroni *post-hoc* test). **c** *In vitro* kinase assays using purified wildtype or G2019S mutant flag-tagged LRRK2 and purified wildtype flag-tagged Rab8a in the absence of nucleotide, or loaded with GTPγS or GDP, respectively. ^33^P incorporation was revealed by autoradiography (left panel), and total protein loading by Coomassie Brilliant Blue staining (right panel) of the same PVDF membrane. **d** Quantification of ^33^P incorporated into Rab8a as compared to total Rab8a from 10 independent experiments. Bars represent mean ± s.e.m; ***, p < 0.001 (two-way ANOVA with Bonferroni *post-hoc* test). **e** Cells were transfected with the indicated flag-tagged Rab8a constructs, and extracts (20 μg) analyzed by Western blotting with an anti-flag antibody, and tubulin as loading control. **f** GTP binding for Rab8a proteins or β-glucoronidase (Gus) was measured by loading with radiolabelled ^3^H-GTP, and normalized to total protein levels. Bars represent mean ± s.e.m. (n=3 experiments); **, p < 0.01. **g** GDP binding for Rab8a proteins or β-glucoronidase (Gus) was measured by loading with radiolabelled ^3^H-GDP, and normalized to total protein levels. Bars represent mean ± s.e.m. (n=3 experiments); **, p < 0.01. **h** GTP retention assay from different Rab8a variants as indicated. Purified proteins were incubated with ^3^H-GTP, a one hundred-fold molar excess of cold GTP was added, and dissociation of nucleotide was measured as a fraction of initial binding at time 0. Data points represent mean ± s.d. (n=3 experiments). Statistical significance was estimated by two-way ANOVA for time versus mutation with Tukey´s *post-hoc* test for comparison to wildtype protein at each time point. **i** GDP retention assay from different Rab8a variants as indicated. Purified proteins were incubated with ^3^H-GDP, a one hundred-fold molar excess of cold GDP was added, and dissociation of nucleotide was measured as a fraction of initial binding at time 0. Data points represent mean ± s.d. (n=3 experiments). Statistical significance was estimated by two-way ANOVA for time versus mutation with Tukey´s *post-hoc* test for comparison to wildtype protein at each time point.
