# Supplementary material for: Parkinson disease-associated mutations in LRRK2 cause centrosomal defects via Rab8a phosphorylation
Source: Mol Neurodegener. 2018 Jan 23;13:3. doi: 10.1186/s13024-018-0235-y (PMC5778812; doi:10.1186/s13024-018-0235-y)
Supplement: Supplementary file 4 — Differential interactions of wildtype and phospho-mimetic Rab8a mutants with GDI1/2 and Rabin8, effects on centrosome splitting and subcellular localization. (DOCX 824 kb) [file 13024_2018_235_MOESM4_ESM.docx]

**
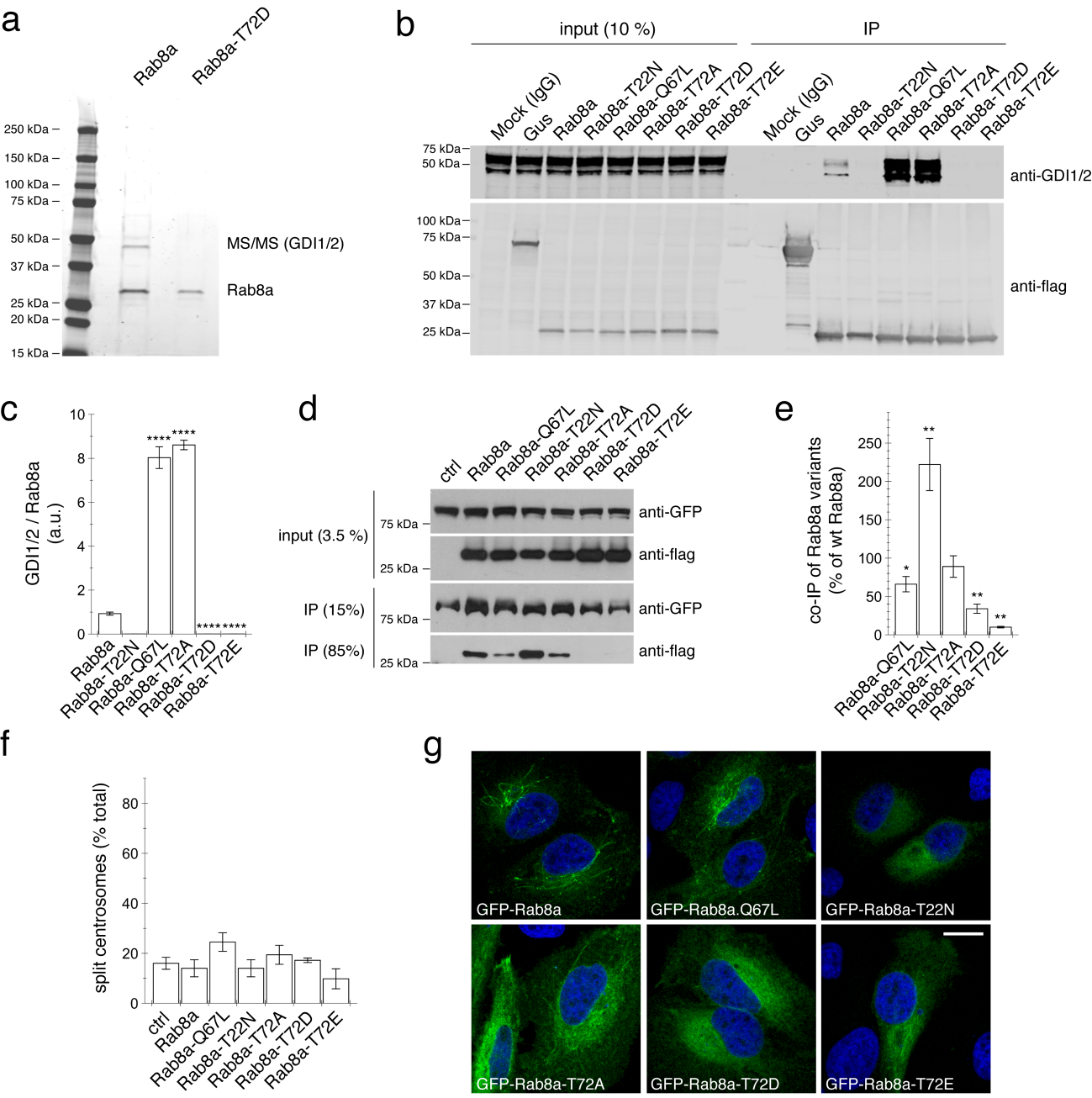
**

**Additional file 4: Figure S4.** Differential interactions of wildtype and phospho-mimetic Rab8a mutants with GDI1/2 and Rabin8, effects on centrosome splitting and subcellular localization. **a** Flag-tagged wildtype and mutant Rab8a were immunoprecipitated from HEK293T cells, followed by SDS-PAGE and Commassie Brilliant Blue staining. The band around 50 kDa co-purifying with wildtype Rab8a was excised from the gel and identified by LC-MS/MS as Rab GDP dissociation inhibitor alpha (GDI1) and beta (GDI2). **b** HEK293T cells were either non-transfected (mock), or transfected with flag-tagged β-glucoronidase (Gus) or Rab8a constructs as indicated, proteins immunoprecipitated with an anti-flag antibody, and immunoprecipitates blotted for endogenous GDI1/2 (upper panel) or flag-tagged proteins (lower panel). **c** Quantification of the amount of GDI1/2 relative to Rab8a in immunoprecipitated complexes from the type of experiments as depicted in B. Bars represent mean ± s.e.m. (n=3 experiments); ****, p < 0.0001 (versus wildtype Rab8a). **d** Cells were transfected with GFP-Rabin8 and either empty vector (ctrl) or different Rab8a variants as indicated. Whole-cell lysates (1 mg) were subjected to immunoprecipitation with a polyclonal anti-GFP antibody followed by immunoblotting with a monoclonal anti-GFP antibody or an anti-flag antibody as indicated, and input was probed for expression levels of GFP-Rabin8 and the different flag-tagged Rab8a constructs. **e** Quantification of the type of experiments depicted in d, normalized to the amount of input, and the amount of coimmunoprecipitation (co-IP) of wildtype Rab8a. Bars represent mean ± s.e.m. (n=3 experiments); ** p < 0.01; * p < 0.05. **f** Quantification of the split centrosome phenotype in either non-transfected HEK293T cells (ctrl) or cells expressing the indicated Rab8a variants. Bars represent mean ± s.e.m. (n=3 experiments). **g** Example of HELA cells expressing the indicated GFP-tagged Rab8a constructs, and stained with DAPI. Scale bar, 15 μm.
