# Supplementary material for: Parkinson disease-associated mutations in LRRK2 cause centrosomal defects via Rab8a phosphorylation
Source: Mol Neurodegener. 2018 Jan 23;13:3. doi: 10.1186/s13024-018-0235-y (PMC5778812; doi:10.1186/s13024-018-0235-y)
Supplement: Supplementary file 5 — Golgi dispersal/disruption has no effect on LRRK2-mediated pericentrosomal/centrosomal accumulation of Rab8a. (DOCX 1670 kb) [file 13024_2018_235_MOESM5_ESM.docx]

**
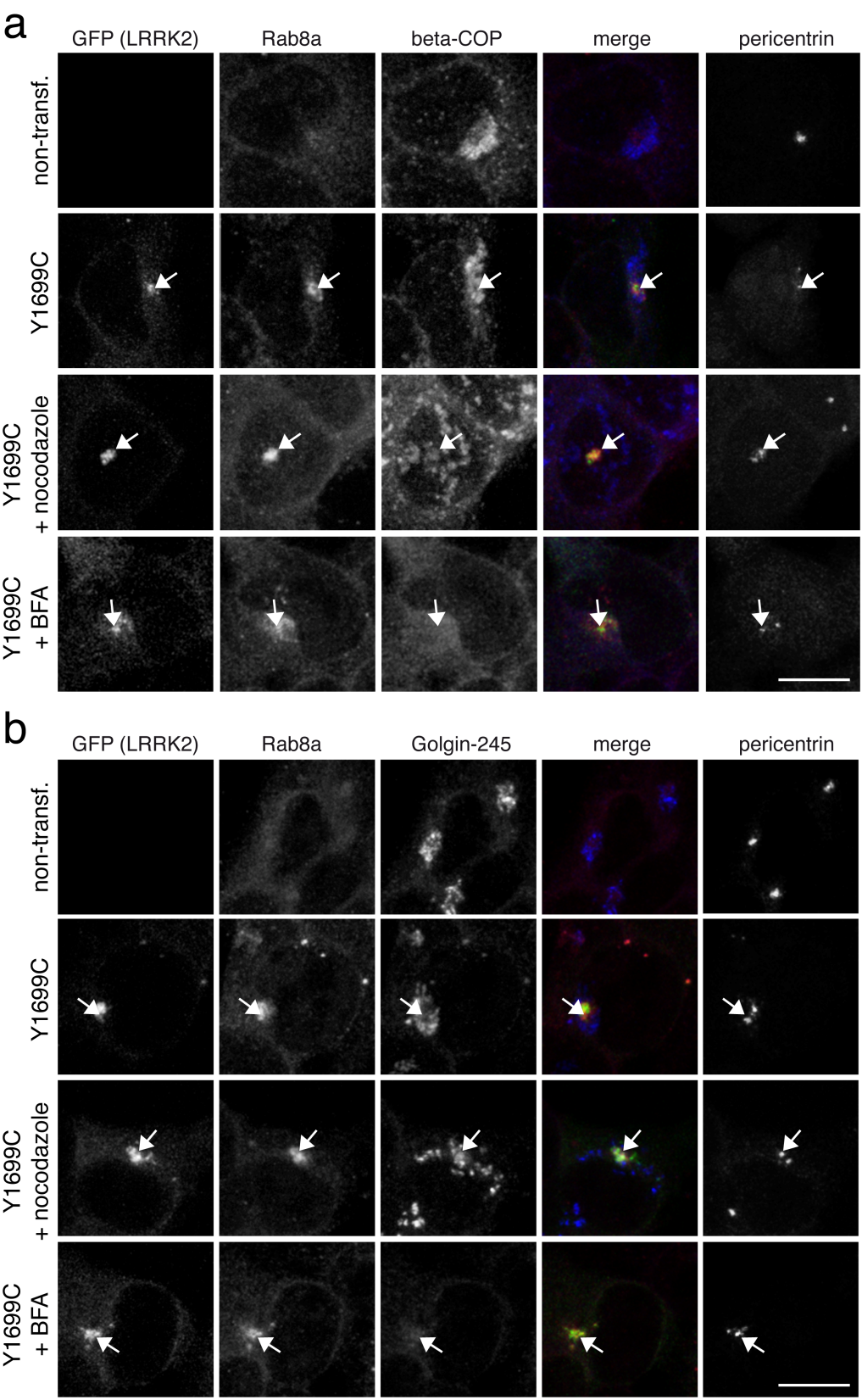
**

**Additional file 5: Figure S5.** Golgi dispersal/disruption has no effect on LRRK2-mediated pericentrosomal/centrosomal accumulation of Rab8a. **a** Example of control cells (non-transf.) or pathogenic LRRK2-transfected cells, either left untreated or treated with nocodazole (200 nM, 2 h) or brefeldin A (BFA, 5 μg/ml, 2h) as indicated, and stained with antibodies against Rab8a, the Golgi marker β-COP, and pericentrin as indicated. Scale bar, 10 μm. **b** Same as in a, but stained with Golgi marker Golgin-245. Scale bar, 10 μm.
