# Supplementary material for: Parkinson disease-associated mutations in LRRK2 cause centrosomal defects via Rab8a phosphorylation
Source: Mol Neurodegener. 2018 Jan 23;13:3. doi: 10.1186/s13024-018-0235-y (PMC5778812; doi:10.1186/s13024-018-0235-y)
Supplement: Supplementary file 6 — Rab8a protein levels and pericentrosomal/centrosomal accumulation of phosphorylated Rab8a in lymphoblasts from control and G2019S mutant LRRK2 PD patients. (DOCX 636 kb) [file 13024_2018_235_MOESM6_ESM.docx]

**
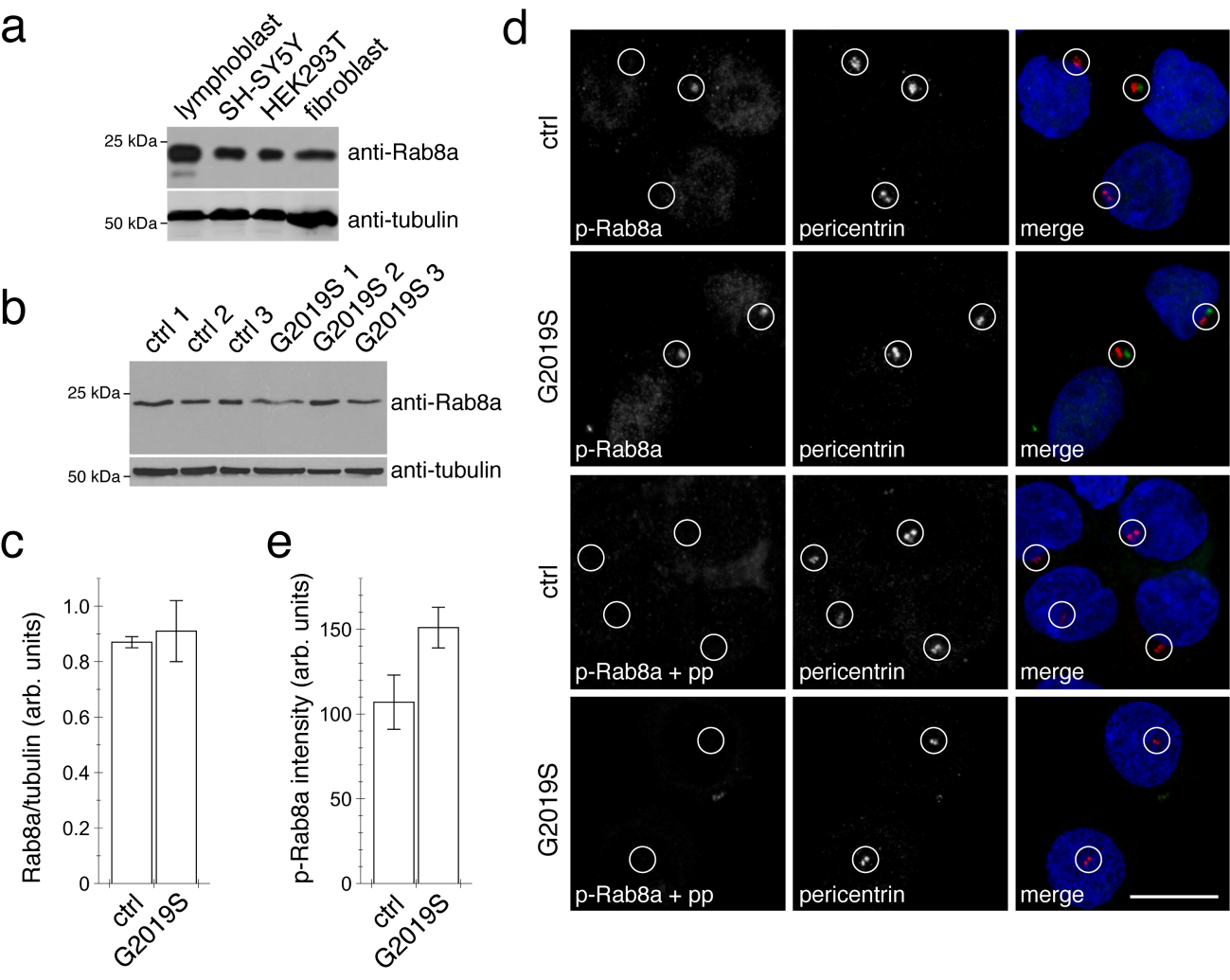
**

**Additional file 6: Figure S6.** Rab8a protein levels and pericentrosomal/centrosomal accumulation of phosphorylated Rab8a in lymphoblasts from control and G2019S mutant LRRK2 PD patients. **a** Extracts (20 μg protein each) from the indicated cells were resolved by SDS-PAGE and blotted with antibodies against Rab8a or tubulin as loading control. **b** Extracts (20 μg protein each) from control and G2019S mutant LRRK2-PD patient lymphoblasts were resolved by SDS-PAGE and blotted with antibodies against Rab8a or tubulin as loading control. **c** Quantification of experiment depicted in b, normalizing Rab8a protein levels to tubulin. **d** Examples of control or G2019S mutant LRRK2-PD patient lymphoblasts stained with phospho-Rab8a antibody preabsorbed with dephosphopeptide (p-Rab8a), or preabsorbed with phosphopeptide (p-Rab8a + pp) as indicated, and stained with centrosomal marker (pericentrin) and DAPI. Scale bar, 10 μm. **e** Quantification of fluorescence intensity of phospho-Rab8a staining in healthy control or LRRK2 mutant PD lymphoblasts. Fluorescence intensity was measured in a circle of 3 μm diameter around individual centrosomes as defined by pericentrin staining, and fluorescence intensity from 30-50 individual cells quantified per cell line (three control, three LRRK2 G2019S mutant cell lines).
