# Supplementary material for: Parkinson disease-associated mutations in LRRK2 cause centrosomal defects via Rab8a phosphorylation
Source: Mol Neurodegener. 2018 Jan 23;13:3. doi: 10.1186/s13024-018-0235-y (PMC5778812; doi:10.1186/s13024-018-0235-y)
Supplement: Supplementary file 7 — Detection of phospho-Rab8a in pathogenic LRRK2-expressing cells as well as in cells co-transfected with wildtype LRRK2 and wildtype Rab8a, but not phospho-deficient Rab8a. (DOCX 958 kb) [file 13024_2018_235_MOESM7_ESM.docx]

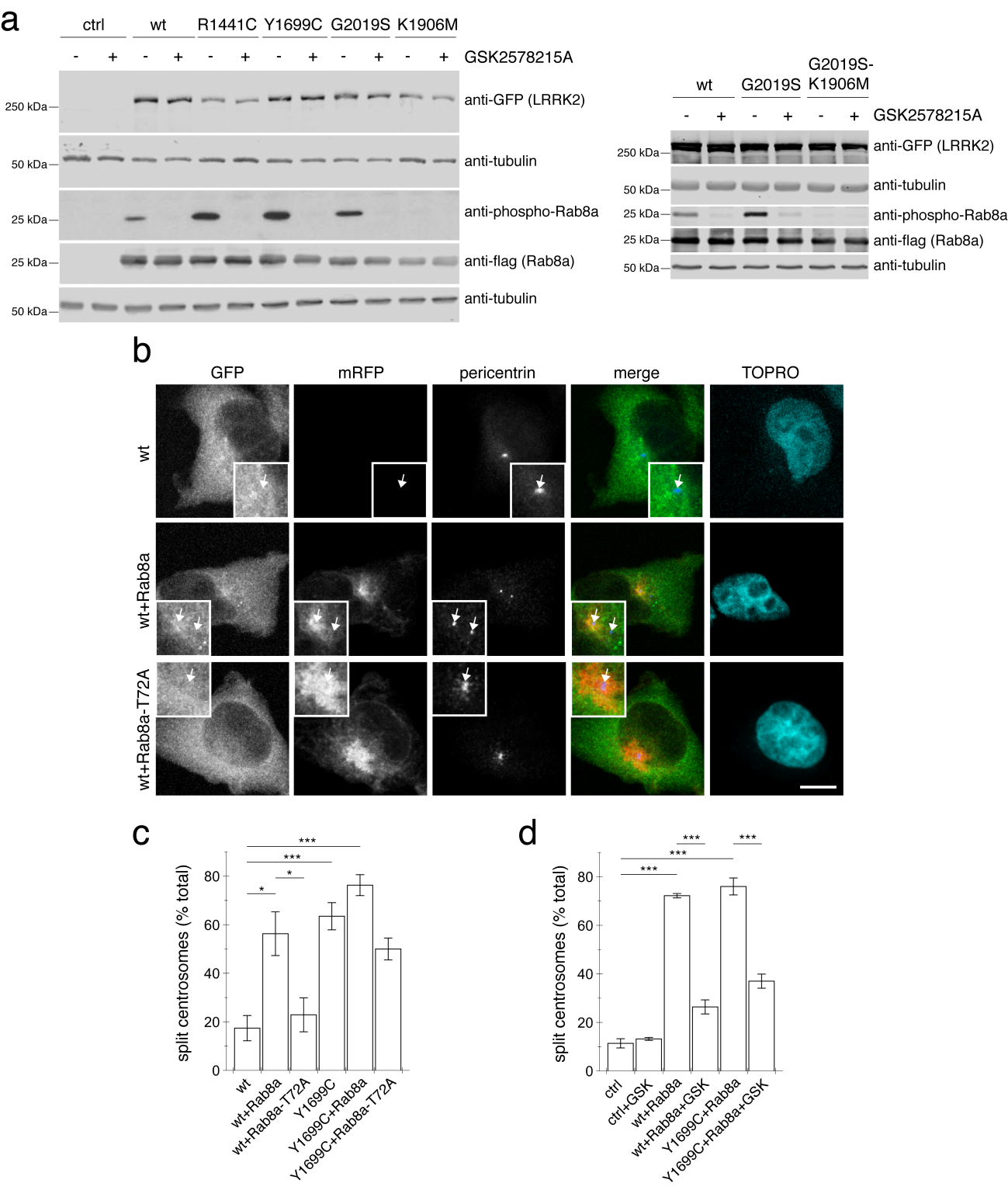


**Additional file 7: Figure S7.** Detection of phospho-Rab8a in pathogenic LRRK2-expressing cells as well as in cells co-transfected with wildtype LRRK2 and wildtype Rab8a, but not phospho-deficient Rab8a. **a** Cells were transfected with constructs as indicated, and cellular lysates subjected to Western blotting with an anti-phospho-Rab8a antibody. Anti-GFP and anti-flag antibodies were used to measure total LRRK2 and Rab8a levels. **b** Example of HEK293T cells co-transfected with GFP-tagged wildtype LRRK2 and either mRFP-Rab8a or mRFP-Rab8a-T72A as indicated, and stained with pericentrin antibody and TOPRO. Scale bar, 5 μm. **c** Quantification of the split centrosome phenotype in HEK293T cells co-expressing wildtype or pathogenic mutant LRRK2 and wildtype or phospho-deficient Rab8a mutant as indicated. Bars represent mean ± s.e.m. (n=3 independent experiments); *** p < 0.005; * p < 0.05. **d** Quantification of the split centrosome phenotype in non-transfected HEK293T cells (ctrl), or in cells co-expressing wildtype or pathogenic mutant LRRK2 along with Rab8a, in either the presence or absence of 500 nM GSK2578215A for 1h as indicated. Bars represent mean ± s.e.m. (n=3 independent experiments); *** p < 0.005.
